# A multidrug ABC transporter with a taste for GTP

**DOI:** 10.1038/s41598-018-20558-z

**Published:** 2018-02-02

**Authors:** Cédric Orelle, Claire Durmort, Khadija Mathieu, Benjamin Duchêne, Sandrine Aros, François Fenaille, François André, Christophe Junot, Thierry Vernet, Jean-Michel Jault

**Affiliations:** 1 University of Lyon, CNRS, UMR5086 “Molecular Microbiology and Structural Biochemistry”, IBCP, 7 Passage du Vercors, F-69367 Lyon, France; 2 Institut de Biologie Structurale (IBS), University Grenoble Alpes, CEA, CNRS, 38044 Grenoble, France; 3 CEA, Institut Joliot, Service de Pharmacologie et d’Immunoanalyse, UMR 0496, Laboratoire d’Etude du Métabolisme des Médicaments, MetaboHUB-Paris, Université Paris Saclay, F-91191 Gif-sur-Yvette cedex, France; 4 Laboratoire Stress Oxydant et Détoxication (LSOD), Institute for Integrative Biology of the Cell (I2BC), CEA, CNRS, Univ Paris-Sud, Université Paris-Saclay, F-91198 Gif-sur-Yvette cedex, France

## Abstract

During the evolution of cellular bioenergetics, many protein families have been fashioned to match the availability and replenishment in energy supply. Molecular motors and primary transporters essentially need ATP to function while proteins involved in cell signaling or translation consume GTP. ATP-Binding Cassette (ABC) transporters are one of the largest families of membrane proteins gathering several medically relevant members that are typically powered by ATP hydrolysis. Here, a *Streptococcus pneumoniae* ABC transporter responsible for fluoroquinolones resistance in clinical settings, PatA/PatB, is shown to challenge this concept. It clearly favors GTP as the energy supply to expel drugs. This preference is correlated to its ability to hydrolyze GTP more efficiently than ATP, as found with PatA/PatB reconstituted in proteoliposomes or nanodiscs. Importantly, the ATP and GTP concentrations are similar in *S*. *pneumoniae* supporting the physiological relevance of GTP as the energy source of this bacterial transporter.

## Introduction

Chemical energy is required to sustain life since any cellular task such as DNA maintenance or replication, translation, cell signaling or transport is an energy-driven process. Many proteins have thus been shaped to harness the chemical energy provided by hydrolysis of the β-γ phosphate bond of a nucleotide, mainly ATP or GTP, to mediate their dedicated function^1^. They predominantly belong to one of the most ancient protein super-families, the P-loop NTPases^2,3^, and their overwhelming presence in all species, between 10 to 18% of each proteome^4^, reflects their versatile and pivotal functions in many cellular pathways^5^. These proteins contain two specific motifs in their sequences: the Walker A motif^6^, G/AX_4_GKT/S, involved in the proper positioning of the polyphosphate moiety of ATP/GTP, and the Walker B motif. This latter is less evident to notice from the sequence because it contains only a conserved aspartate, located at the end of a hydrophobic β-strand, involved in the coordination of a magnesium ion required for catalysis^7^.

Some of the P-loop NTPases hydrolyze exclusively GTP because they bear an additional motif, the N/TKXD sequence, which mainly dictates a strong specificity for the guanine, as exemplified by the Ras protein^7^. In contrast, P-loop ATPases are generally more promiscuous as they can often hydrolyze other nucleotides such as GTP or ITP (e.g. the F1-ATPase^8^), albeit at a lower rate than ATP. Yet, this relative lack of specificity may mainly operate *in vitro*, in particular due to the abundance of ATP as compared to other nucleotides in different organisms^9–11^. This seeming lack of specificity of many ATPases is due to limited contact with the base moiety of the nucleotide, often restricted to a stacking interaction between the adenine ring and an aromatic residue^1^.

The ABC (ATP-Binding Cassette) transporters family is part of the P-loop ATPases super-family and is involved in the vectorial transport, import or export, of a huge variety of compounds including ions, sugars, lipids, peptides and large hydrophobic molecules. The dysfunction of prominent ABC transporters is responsible for severe pathologies such as cystic fibrosis, tangier disease and adrenoleukodystrophy, while the subversion of multidrug ABC transporters confers resistances to therapeutic treatments in malignant cells or pathogenic microorganisms^12,13^. These transporters share a common topology with two transmembrane domains (TMDs) and two nucleotide-binding domains (NBDs). Besides Walker A and B motifs, the NBDs contains several additional motifs involved in ATP binding, including the signature of this family (a stretch of ~12 residues usually starting by LSGGQ)^1^. The A loop is another structural motif that provides an aromatic residue to stack the adenine ring^14^. During the catalytic cycle, the two NBDs interact in a transient head-to-tail orientation to form two composite ATP-binding sites at the NBDs interface, each site being formed by all motifs from one NBD except the ABC signature which is provided in *trans* by the other NBD^15,16^. The paradigm concerning the catalytic mechanism of ABC transporters is that they are powered by ATP hydrolysis but, like other P-loop ATPases, some can use other nucleotides such as GTP or UTP, although in a less efficient manner *in vitro*^17–21^. Thus, the physiological relevance of these findings remains unclear.

*Streptococcus pneumoniae* is a life-threatening bacterial pathogen that causes otitis, pneumonia, meningitis and sepsis, despite the availability of vaccines which are, nevertheless, poorly efficient towards new emerging serotypes. It causes more than 1.6 million deaths worldwide each year according to the World Human Organization that has recently included drug-resistant *Streptococcus pneumoniae* in its priority pathogens list for research and development of new antibiotics. Importantly, the multidrug ABC transporter, PatA/PatB, has been shown to be involved in the resistance of *S*. *pneumoniae* to fluoroquinolones in clinical settings^22,23^ and we have shown that both subunits are required to form a functional heterodimeric multidrug transporter *in vitro*^24^.

Here, we show that when GTP is used instead of ATP, the drug transport activity of PatA/PatB is greatly enhanced, in particular at 37 °C. This is related to the unusual ability of PatA/PatB to preferentially hydrolyze GTP at this temperature. Importantly, the intracellular concentrations of ATP and GTP are in similar range in *S*. *pneumoniae* and upon exposure to norfloxacin, a drug transported by PatA/PatB, the concentration of GTP dramatically increases whereas that of ATP is essentially unaffected. Thus, PatA/PatB is an ABC transporter that has diverged to favor GTP as the energy source and this is presumably a consequence of *S*. *pneumoniae* lifestyle.

## Results

### Transport activity of PatA/PatB

**Figure 1:**
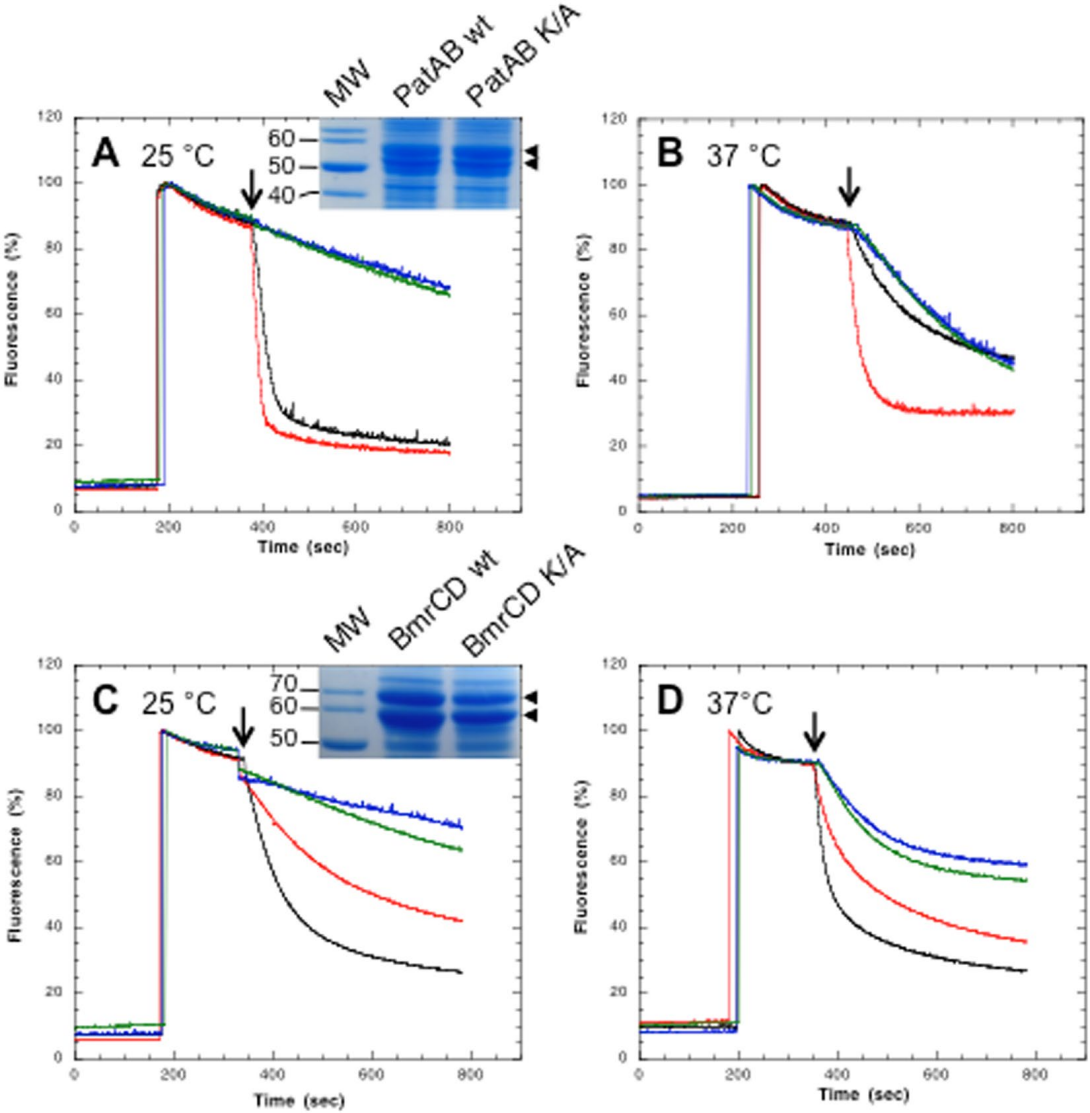
Hoechst transport of PatA/PatB and BmrC/BmrD. Hoechst 33342 (1 µM) was added to inside-out vesicles containing overexpressed wild-type PatA/PatB (50 µg of total proteins; panels A and B) or wild-type BmrC/BmrD (200 µg of total proteins; panels C and D) and transport activities were monitored at 25 °C (**A** and **C**) or 37 °C (**B** and **D**) upon addition of 2 mM ATP (black traces) or 2 mM GTP (red traces), as indicated by the arrows. Data were normalized to 100%. As a control, the inactive Lys to Ala Walker-A double mutants was also used (green and blue traces, in the presence of ATP and GTP, respectively) for PatA/PatB (panels A and B) or BmrC/BmrD (panels C and D). The insets are cropped SDS-PAGE of *E*. *coli* membrane vesicles showing the level of overexpressed wild-type (wt) or double mutants (K/A) for PatA/PatB (panel A) or BmrC/BmrD (panel C; positions are indicated by arrowheads on the right). Original (uncropped) SDS-PAGE are shown in Fig. S2. The sizes of the molecular weight markers (MW) are also indicated of the left (in kDa). A representative experiment of ten (panels A and B) and three independent experiments (panels C and D) is shown here. Depending on the membrane batches, the Hoechst transport specifically catalyzed by PatA/PatB at 37 °C (i.e. corrected from the basal transport displayed by the inactive mutant) is, at least, always 4 fold better when fueled with GTP as compared to ATP.

The heterodimer PatA/PatB involved in the resistance to fluoroquinolone was shown previously to transport Hoechst 33342 *in vitro* when inside-out membrane vesicles (IMV) were prepared from *E*. *coli* that contained overexpressed PatA/PatB. Because it belongs to the ABC transporter family, PatA/PatB was classically energized with ATP to measure its transport activity^24^. However, a comparison of the hydrolytic properties of PatA/PatB solubilized by lauryl maltose neopentyl glycol (LMNG) and purified in this detergent revealed that it had a much higher GTPase activity (~2.5 fold) as compared to its ATPase activity (Fig. S1, and see below). We determined the dissociation constant of the fluorescent analogues TNP-GTP and TNP-ATP as 5.6 ± 1.3 μM and 3.4 ± 1.1 μM, respectively, suggesting that the lower ATPase activity was not due to a decrease in nucleotide affinity. Therefore, we decided to investigate the effect of these two nucleotides on the Hoechst 33342 transport by PatA/PatB. At 25 °C, both ATP and GTP were capable to fuel PatA/PatB to efflux the Hoechst compound, but GTP was consistently more efficient than ATP (Fig. 1A and Fig. S3 for close-up view). In order to rule out that the high transport rate observed with GTP was due to a contaminant, an inactive mutant with the invariant lysine of the Walker-A motif of both PatA and PatB subunits substituted by an alanine was used. This mutant was overexpressed and addressed to the membrane similarly to the wild-type transporter (Fig. 1A and Fig. S2 for whole SDS-PAGE), as previously reported^24^. However, in contrast to the wild-type, it was unable to transport the Hoechst in the presence of either ATP or GTP (Fig. 1A; and see Fig. S4 for raw, i.e. unnormalized, data). Because *S*. *pneumoniae* colonize different environments in terms of temperature, sources of nutrients and energy throughout its lifestyle^25^, transport activity were also measured at 37 °C. This is in fact the temperature that the bacteria have to face upon infection of deep tissues in its human host (e.g. lungs, bloodstream and spleen)^26^. Unexpectedly, a more dramatic effect was observed at 37 °C. GTP proved to be quite superior to ATP to power the efflux of Hoechst as accumulation of this dye in the lumen of the vesicles reached a plateau within few seconds (Fig. 1B). In fact, the ATP-dependent Hoechst transport catalyzed by PatA/PatB was only barely, yet significantly, above the background transport exhibited by the inactive mutant. The residual Hoechst transport activity observed at this temperature with the inactive mutant is presumably coming from an endogenous multidrug *E*. *coli* transporter mainly active at this temperature, as it is also detected with the control ‘empty’ vesicles (Fig. S5).

**Figure 2:**
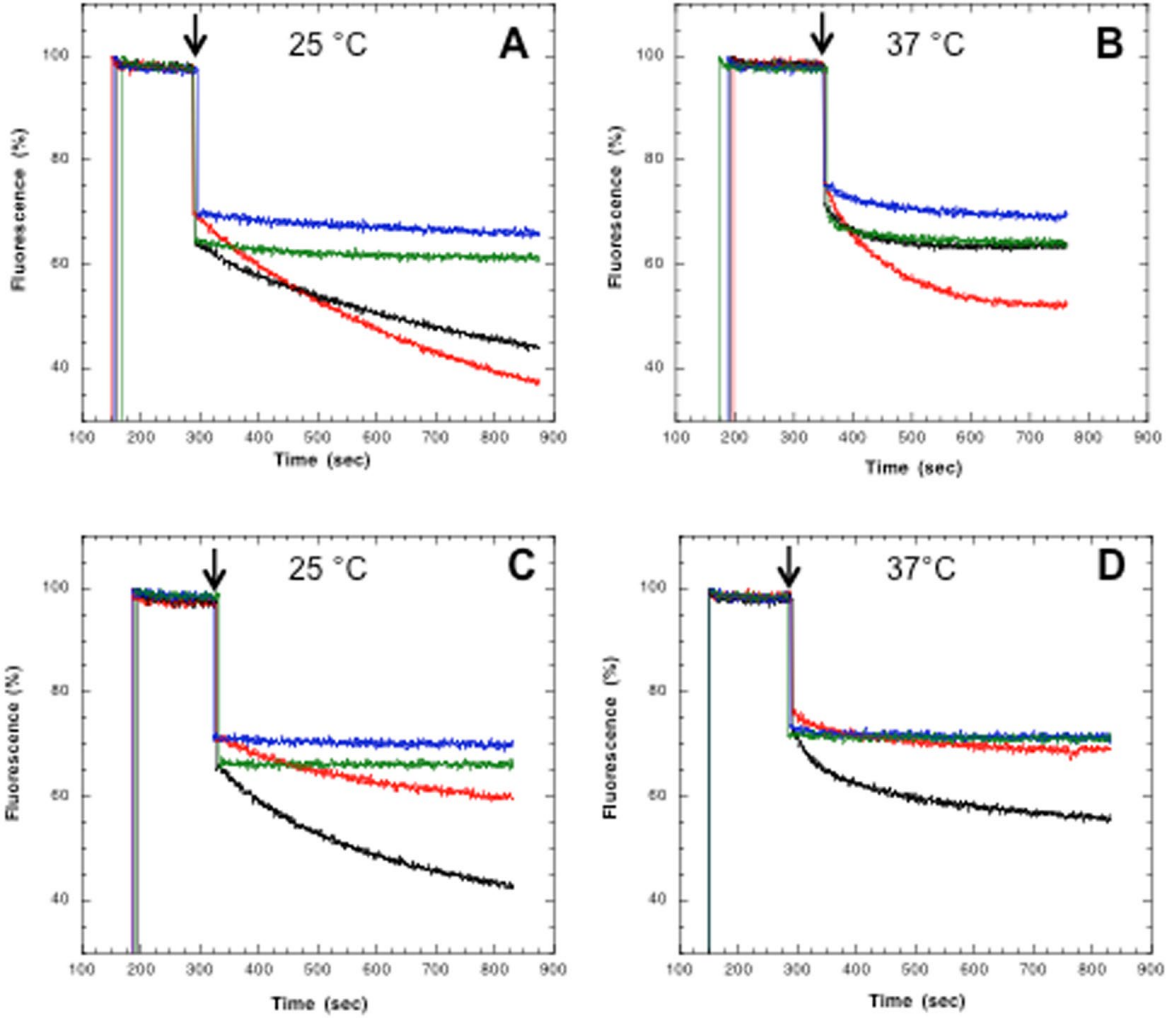
Doxorubicin transport of PatA/PatB and BmrC/BmrD. The experimental conditions were similar to those used in Fig. 1 except that 2 µM of Doxorubicin was added to inside-out vesicles containing overexpressed wild-type PatA/PatB (panels A and B, 50 and 100 µg of total proteins, respectively) or wild-type BmrC/BmrD (panels C and D, 200 µg of total proteins) and transport activity was monitored at 25 °C (**A** and **C**) or 37 °C (**B** and **D**) upon addition of 2 mM ATP (black traces) or 2 mM GTP (red traces), as indicated by the arrows. Data were normalized to 100%. The inactive Lys to Ala Walker-A double mutants were also used (green and blue traces, in the presence of ATP and GTP, respectively). The initial jump of fluorescence following nucleotide addition is due to the nucleotide inner effect filter at the wavelength used here^51^. A representative experiment of triplicates is shown here.

**Figure 3:**
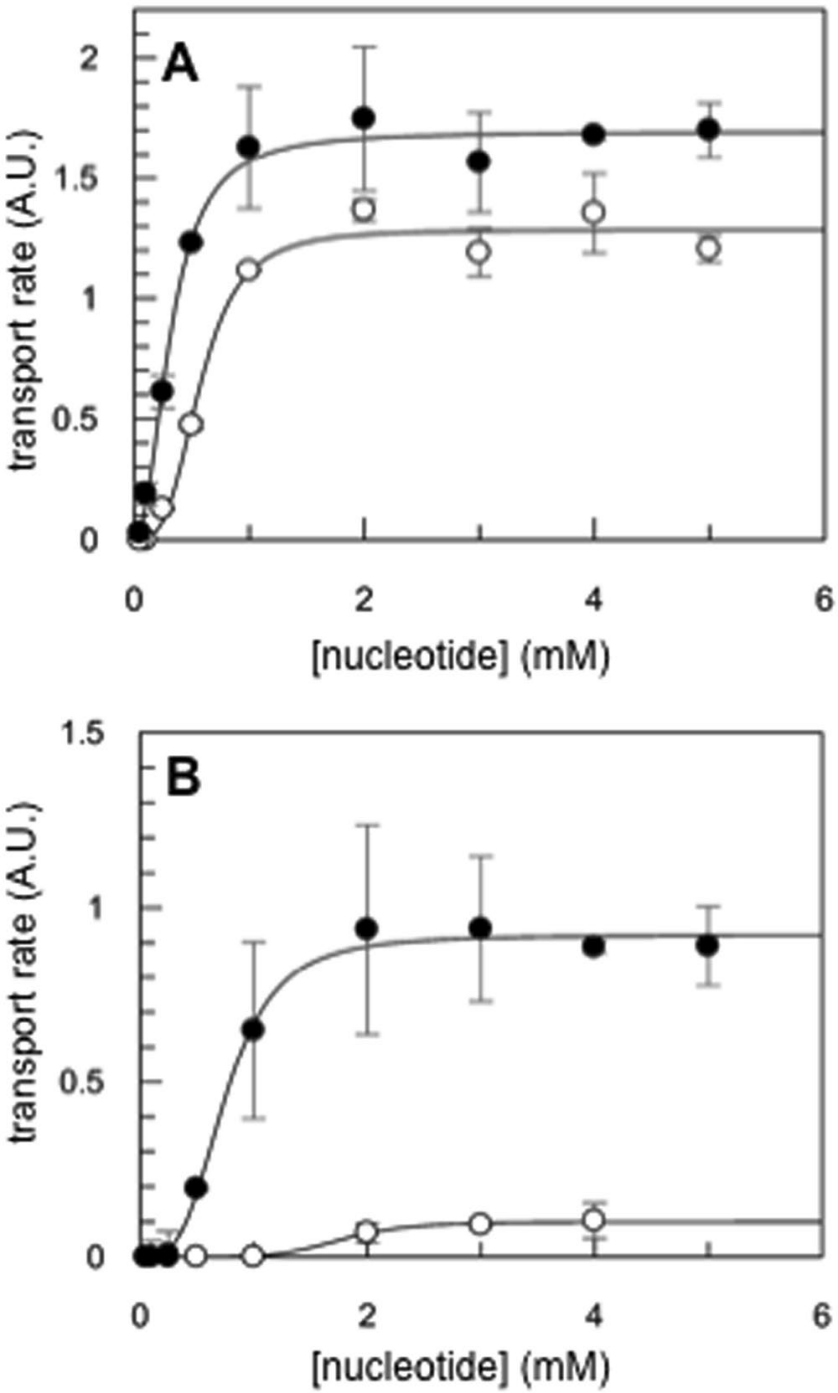
Effect of nucleotide concentration on the Hoechst transport activity of PatA/PatB. 1 µM Hoechst was added to inside-out vesicles containing overexpressed wild-type PatA/PatB or PatA/PatB Walker A double mutant (50 µg of total membrane protein each). The initial transport rates were measured just after the addition of ATP (white dots) or GTP (black dots) at 25 °C (**A**) or 37 °C (**B**) and the values obtained for the wild-type were corrected by those obtained with the double mutant. A representative experiment of two separate experiments is shown here. Triplicates were realized for each nucleotide concentration using the same batch of inside-out vesicles preparation and the error bars represent the standard deviation of the mean.

Puzzled by these results with PatA/PatB, we compared the transport activity of a related drug transporter from *Bacillus subtilis*, BmrC/BmrD^27^ (Tables S1 and S2). Clearly, BmrC/BmrD behaves as a conventional ABC transporter being more active in the presence of ATP as compared to GTP as previously observed for other transporters^17,19^, regardless of the temperature of the assay (Fig. 1C and D; see Fig. S4 for raw, i.e. unnormalized, data). An inactive mutant, (same mutations introduced as in the PatA/PatB double mutant), also showed no Hoechst transport at 25 °C, while a significant transport activity still remained at 37 °C. Again, this reflects presumably the activity of an endogenous *E*. *coli* transporter that functions mostly at 37 °C. In order to check if the preference of PatA/PatB for GTP was independent of the transported drug, doxorubicin was also used. In multidrug ABC transporters, doxorubicin and Hoechst 33342 have been shown to bind to two different sites^28,29^. With GTP as the energy source, the rate of doxorubicin transport by PatA/PatB was faster at 25 °C and at 37 °C as well (Fig. 2A and B, red curves; see Fig. S6 for raw, i.e. unnormalized data). In contrast, ATP was the preferred energy substrate for BmrC/BmrD at both temperatures (Fig. 2C and D; see Fig. S6 for raw, i.e. unnormalized, data). No doxorubicin transport was detected when the inactive PatA/PatB or BmrC/BmrD mutants were used (Fig. 2) or in the presence of vanadate (Vi), a classical inhibitor of ABC transporters^30^ (Fig. S7). Other nucleotides were used to check if PatA/PatB was promiscuous for its energy supply. Apart from ITP and TTP that allowed some transport activity at 25 °C, albeit much less efficiently than GTP, other nucleotides were unable to power the Hoechst transport of PatA/PatB (Fig. S8). The preference for GTP was even more pronounced at 37 °C where ITP, and to a lower extent ATP, allowed some transport (∼ 35% and ~20%, respectively). A possible explanation for the reduced efficacy of ATP as compared to GTP would be that PatA/PatB has a reduced affinity for the former nucleotide in membrane context. To address this question, the transport rate was measured with increasing nucleotide concentrations. The maximal rate of Hoechst transport was reached at a similar nucleotide concentration (~2 mM) for both ATP and GTP at 25 °C (Fig. 3A). The same trend was observed at 37 °C for both nucleotides (Fig. 3B) but in the presence of ATP, a close to background rate of transport was observed for the wild-type PatA/PatB regardless of the nucleotide concentration used. This showed that ATP was hardly able to power this transporter at this temperature. Overall, these results emphasize that GTP is the genuine energy fuel of PatA/PatB *in vitro* regardless of the drug to be effluxed, and it is almost exclusively used at higher temperature.

### ATPase and GTPase activities of PatA/PatB

**Figure 4:**
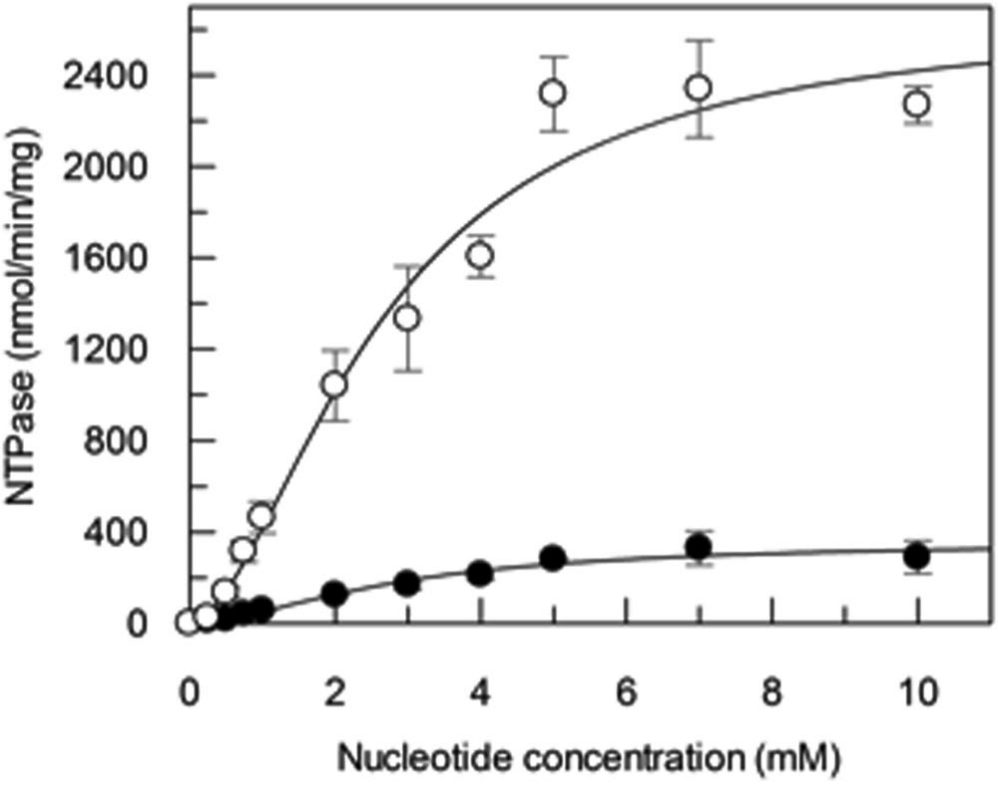
NTPase activities of PatA/PatB reconstituted into proteoliposomes. The ATPase and GTPase activities of purified PatA/PatB reconstituted into proteoliposomes were measured at 37 °C and are shown in black and white symbols, respectively. Data are the average of two separate experiments, each of them being performed with a different batch of purified PatA/PatB. Error bars represent the standard deviation of the mean. The fitted parameters obtained with GraFit 7.0 are *V*_max_ 356 ± 39 nmol/min/mg proteins, *K*_M_ 2.83 ± 0.5 mM and n_H_ 1.73 ± 0.34 for the ATPase activity and *V*_max_ 2660 ± 279 nmol/min/mg proteins, *K*_M_ 2.64 ± 0.46 mM and n_H_1.74 ± 0.33 for the GTPase activity.

**Figure 5:**
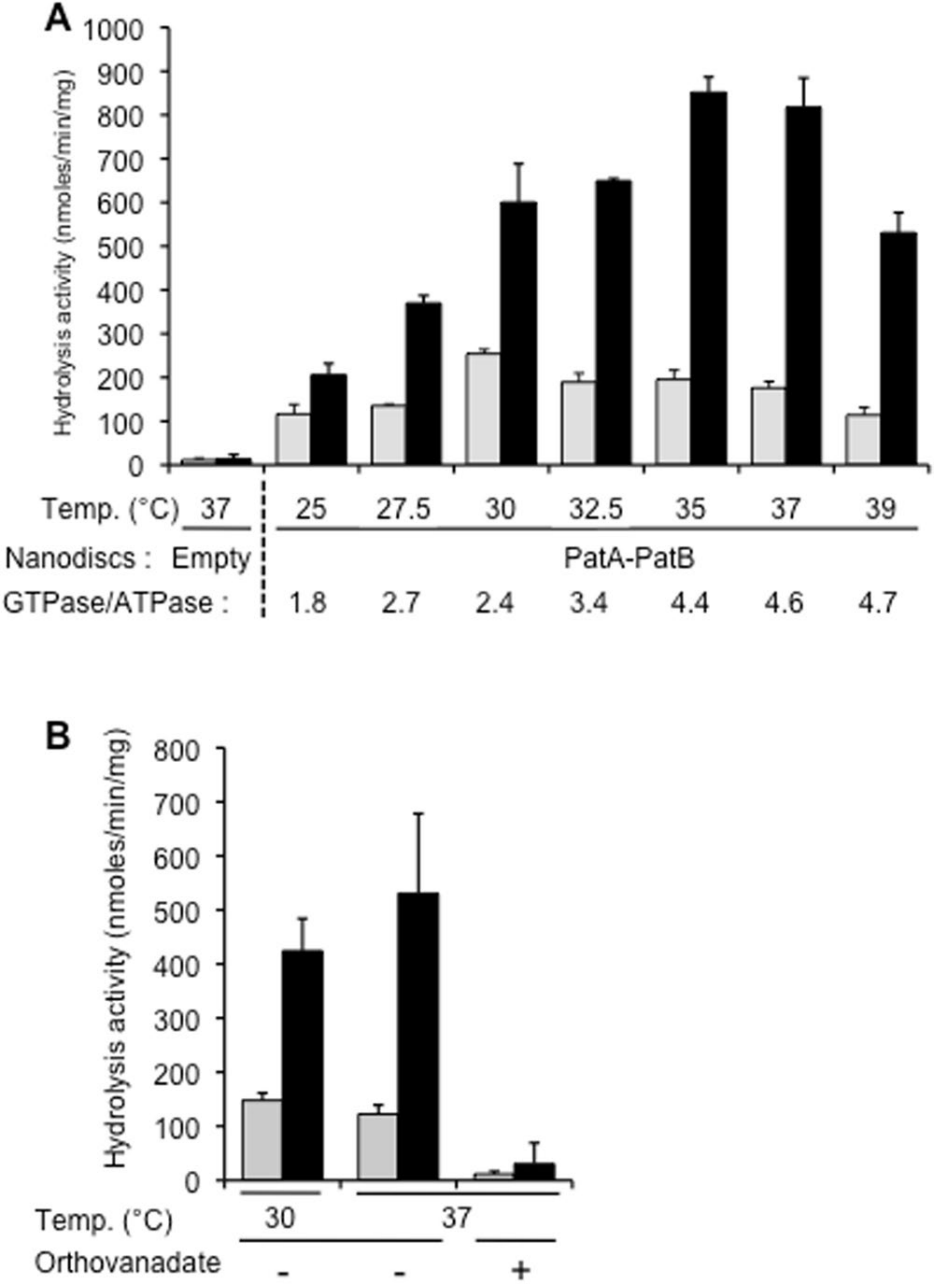
Effect of the temperature on NTP hydrolysis activities of PatA/PatB reconstituted into nanodiscs. Hydrolysis activity of PatA/PatB (4 µg) incorporated into nanodiscs was measured at 340 nm upon addition of 4 mM ATP (grey bars) or 4 mM of GTP (black bars). Error bars represent the standard deviation of the mean for three independent measurements. (**A**) Nanodiscs were prepared from *E*. *coli* lipids and NTPase activities were determined at the indicated temperatures. (**B**) Nanodiscs were prepared from *S*. *pneumoniae* lipids and NTPase activities were determined at 30 °C and 37 °C with or without 4 mM orthovanadate.

Multidrug ABC transporters are known to possess a high basal ATPase activity even in the absence of a transported drug^29,31^. As mentioned above, detergent purified PatA/PatB exhibited a GTPase activity higher than its ATPase activity (Fig. S1). To avoid any bias resulting possibly from the presence of the detergent, which may alter the native properties of transporters^32^, reconstitutions of PatA/PatB into either proteoliposomes^33^ or nanodiscs^34^ were performed. To this end, membrane fraction containing PatA/PatB were first solubilized in LMNG as this detergent was recently shown to advantageously replaced DDM for solubilization and purification of BmrA^35^ or BmrC/BmrD (unpublished results). Following purification as described before, PatA/PatB was reconstituted into proteoliposomes using *E*. *coli* lipids by removing the detergent with bio-beads^36^, and the kinetic parameters for nucleotide hydrolysis were measured at 37 °C. Regardless of the nucleotide concentration, the GTPase activity remains always higher than the ATPase one, with a *V*_max_ of 2660 ± 279 and 356 ± 39 nmol/min/mg proteins for GTPase and ATPase activities, respectively, and a positive cooperativity was observed in both cases (n_H_ ~ 1.7 for both kinetics; Fig. 4). The apparent *K*_M_ values were in similar ranges, 2.64 ± 0.46 mM for GTPase and 2.83 ± 0.5 mM for ATPase activities. We also studied the NTPase activities of PatA/PatB in the presence of drugs, i.e. Hoechst 33342, doxorubicin, norfloxacin and ciprofloxacin (Fig. S9). Albeit the GTPase activities are reduced at certain drug concentrations, they remained much higher than the ATPase activities at all drug concentrations tested. In addition, purified PatA/PatB was incorporated into nanodiscs. The nanodiscs containing a mixture of empty particles and particles containing incorporated PatA/PatB were separated from the aggregates by size exclusion chromatography (Fig. S10). Gel electrophoresis of the nanodisc fractions showed that it contains both PatA and PatB in a similar amount (two bands above 50 kDa) and also an intense third band at ~25 kDa, the expected molecular weight of the membrane scaffold protein MSP1E3D1 (Fig. S10). This reveals that a significant proportion of empty nanodiscs was still present in these fractions but it had no impact on the further analysis of these particles (see below). Increasing concentrations of purified PatA/PatB were resolved on the same polyacrylamide gel allowing the relative quantification of the PatA/PatB concentration present in the nanodisc preparations. The hydrolytic activities of the nanodiscs containing PatA/PatB were studied at different temperatures, to which the pneumococcus is exposed to during host infection, namely a range of 25 to 39 °C^26^. In contrast to empty nanodiscs which were essentially devoid of either ATPase or GTPase activity at 37 °C, the PatA/PatB containing nanodiscs hydrolyzed efficiently these two nucleotides regardless of the temperature used, but the GTPase activity always remains higher than the ATPase activity (Fig. 5A). At higher temperatures (35–39 °C), the preference for GTP was marked (~4.5 fold) whereas this trait was less pronounced at the lowest temperature (1.8 fold). The optimal temperatures were also different being close to 35–37 °C for the GTPase activity, whereas it was about 30 °C for the ATPase activity.

To reconstitute PatA/PatB in a lipidic environment even closer to its native state, PatA/PatB were incorporated into nanodiscs using lipid extracts prepared from *S*. *pneumoniae* membranes, since its lipid composition differs from that of *E*. *coli* (Table S3). Although the activities were slightly lower than those obtained previously in *E*. *coli* lipids, the profile of nucleotide hydrolysis activities remained similar at either 30 or 37 °C (Fig. 5B), with GTP being more efficiently hydrolyzed than ATP. Addition of orthovanadate strongly reduced nucleotide hydrolysis, with 90% and 94% inhibition for ATPase and GTPase activities, respectively.

### Nucleotide concentrations in *S. pneumoniae*

**Figure 6:**
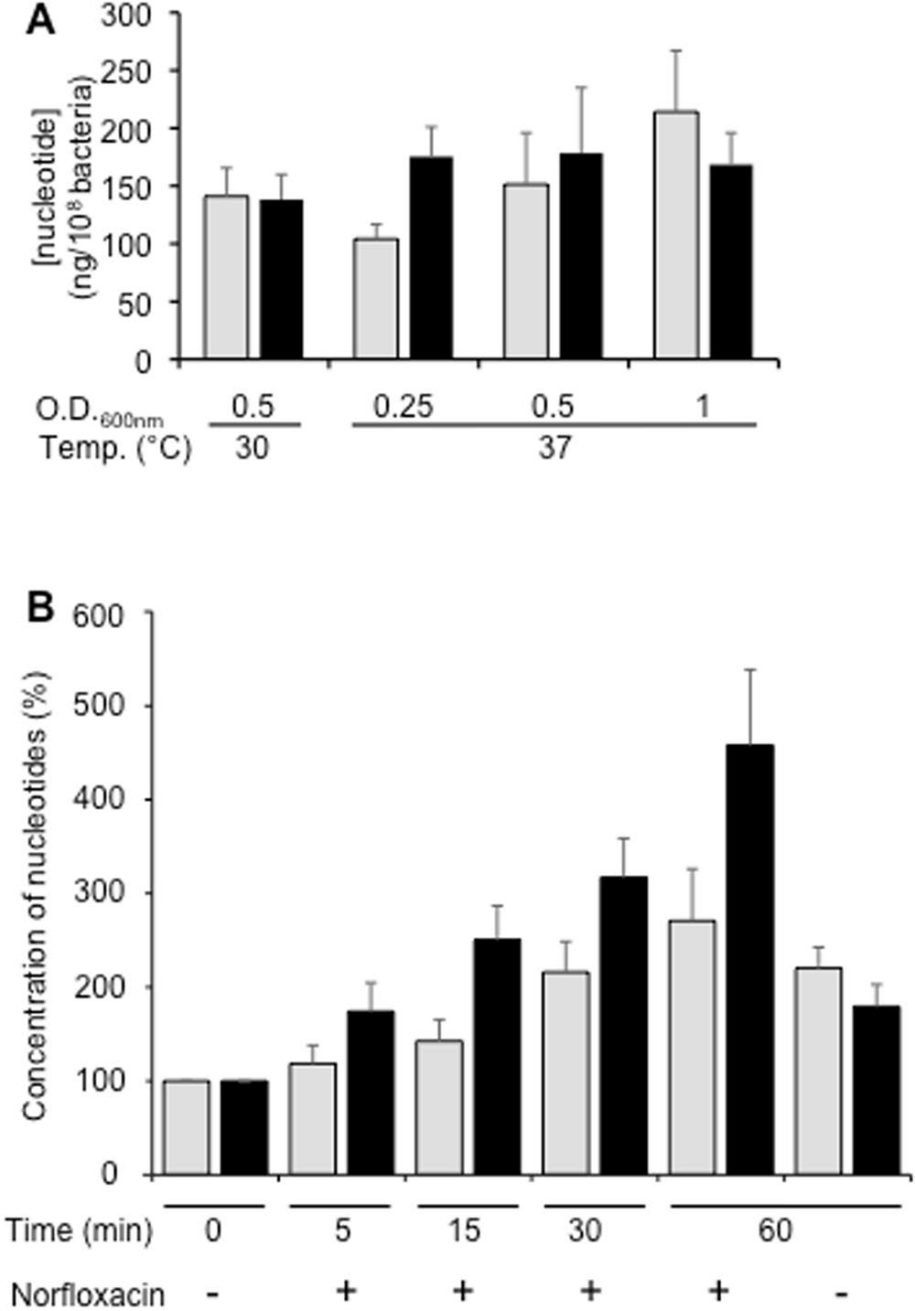
Intracellular concentrations of ATP and GTP. (**A**) *S*. *pneumonia* were grown in TH medium at 30 °C or 37 °C until the indicated absorbance then rapidly filtered and frozen in liquid nitrogen before nucleotides extraction. Concentrations of ATP (grey bars) and GTP (black bars) were determined by mass spectrometry and were expressed in ng per 10^8^ bacteria. Error bars represent the standard deviation of the mean for five separate experiments. (**B**) *S*. *pneumonia* were grown in TH medium at 37 °C and when the absorbance at 600 nm reached 0.25, they were exposed to 16 µg/mL of norfloxacin for the indicated period of time. Nucleotide concentrations were measured in control cultures before exposition to norfloxacin (T0) and after 60 min without exposition (T60 −). Bacteria were prepared as above and concentrations of ATP (grey bars) and GTP (black bars) were determined by mass spectrometry and expressed in % of the non-treated control (T0). The error bars represent the standard deviation of the mean for three (non-treated samples) or four (treated samples) separate experiments.

In order to check if our *in vitro* results could bear a physiological relevance, the *in vivo* concentration of ATP and GTP had to be considered. We thus performed analyses to measure their concentrations by mass spectrometry. *S pneumonia* R6 strain was grown at different growth phases and temperature and its intracellular contents of ATP and GTP were determined. It revealed that both nucleotides were present in similar amounts, ∼150 ng/10^8^ bacteria, at 30 °C (Fig. 6A). Given that the size of pneumococcus for the R6 strain cultured *in vitro* in liquid medium was determined from electron microscopy images to be 878 ± 16.6 nm width and 1680 ± 63 nm length (measured on 48 bacteria), and considering that the shape of pneumococcus is ovoid we estimated that the volume of a single pneumococcus is about 0.68 fL. As a result, intracellular concentrations of both ATP and GTP would be around 4 mM each. A similar trend was observed at 37 °C with a rather constant GTP level regardless of the growth stage (∼175 ng/10^8^ bacteria), while that of ATP varied between 100 and 200 ng/10^8^ bacteria depending on the growth phase.

It was shown previously that PatA/PatB is responsible for resistance to fluoroquinolones, i.e. norfloxacin and ciprofloxacin^24,37^. Importantly, fluoroquinolone treatments *in vitro* led to the emergence of resistant *S*. *pneumoniae* strains with increased expression of PatA/PatB^22^ and overexpression of this transporter was found in clinical isolates resistant to fluoroquinolones^23^. Here, the effect of norfloxacin on the intracellular concentrations of ATP and GTP was evaluated (Fig. 6B). When the absorbance of the cultures reached 0.25, the antibiotic was added, or not, and the growth was pursued for 1 h. This led to a 2.2 fold increase in the ATP level in the control bacteria; this level was not significantly affected by the norfloxacin treatment. However, a different scenario occurred for GTP, with a sharp increase in the GTP concentration upon norfloxacin addition (relatively to the original level) much above the effect measured in the untreated sample. Thus, norfloxacin seems to trigger a specific accumulation of GTP within *S*. *pneumoniae*. To confirm this observation, we also analyzed the GTP intracellular concentration when the cells were incubated with 3 other putative substrates of PatA/PatB^38^. A strong increase in GTP concentration was observed in presence of ciprofloxacin and acriflavin, but not with berberin, while ATP level was not markedly impacted by any of these antibiotics (Fig. S11).

## Discussion

To finely tune all cellular processes in a living cell, a huge amount of chemical energy is constantly needed. Apart from the ion-gradient used by families of secondary transporters to exchange molecules across a membrane, the main energy supply used to power the proteins involved in these processes is mostly provided by the hydrolysis of two nucleotides, ATP or GTP. However, proteins have been tailored to favor either one of these two nucleotides and to transform their chemical energy into mechanical energy so as to fulfill a precise function. For instance, ATP is used by all families of primary transporters involved in exchange with the external milieu or between different cellular compartments (e.g. Ca^2+^-ATPases, H^+^-ATPases…) whereas GTP is notably selected to mediate intracellular signaling or to ensure translation by protein synthesis factors^39^.

**Figure 7:**
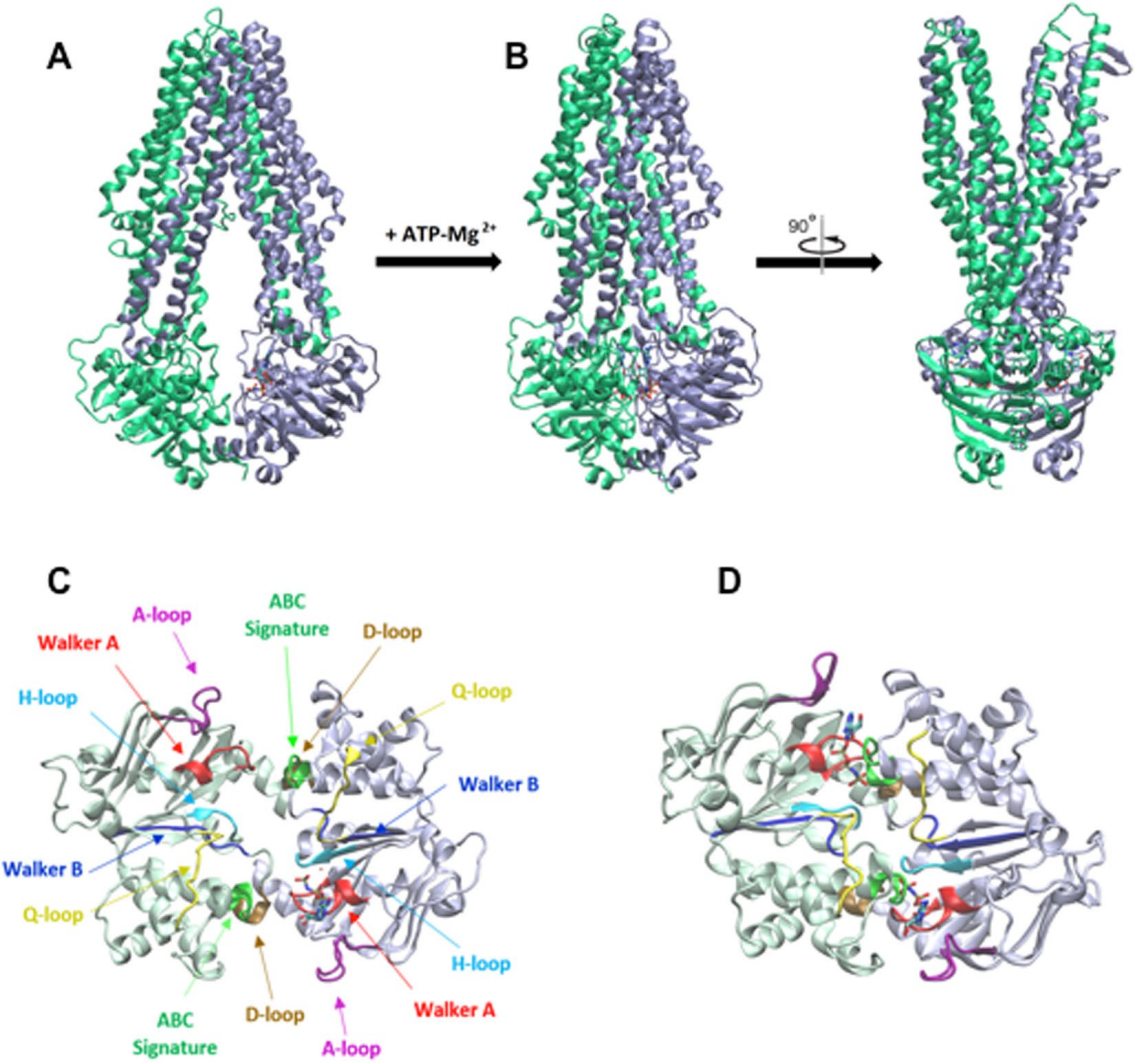
Structural models of PatA/PatB in the inward- and outward-facing conformations. The inward- and outward-facing models of PatA (ice blue) and PatB (green) are respectively based on the crystal structure of TM287/TM288 (PDB code 3QF4^60^) with one AMP-PNP (Adenylylimidodiphosphate) bound to one NBD (**A**), and the crystal structure of Sav1866 (PDB code 2ONJ^61^). In the latter, the two NBDs are closely packed with two bound AMP-PNP sandwiched at their interface (**B**). The NBDs viewed from the top are in the inward-facing conformation (**C**) or outward-facing conformation (**D**). Conserved motifs are colored in red (Walker A), blue (Walker B), green (ABC signature), purple (A-loop), brown (D-loop), cyan (H-loop) and yellow (Q-loop). The figure was prepared using VMD 1.9.3^62^. All the structures are represented in the new cartoon mode and nucleotides are shown in licorice representation in atom coloring mode.

The vast majority of ATPases and GTPases originates from a common ancestor with a P-loop-containing nucleoside triphosphate hydrolase fold, which is the most ancient protein architecture known^2^. This ancestral P-loop protein, bearing both Walker A and B motifs, probably had a low specificity for nucleotides and low catalytic efficiencies^4^. In the course of evolution, a new lineage emerged called ASCE (for ‘additional strand, catalytic E’) because it bears an additional β-strand inserted between the Walker A and B motifs and also it recruited a catalytic glutamate. This shaped a divergent fold with a higher catalytic efficiency together with a preference for ATP^4^. ATPases that belong to this ASCE branch are for instance motor enzymes (e.g. Fo-F1), helicases (e.g. recA) and ABC proteins^1^. Concerning the GTPases lineage, it also evolved by addition of an extra β-strand on one side of the β-sheet core domain but, more importantly, it molded a new NKxD motif that confers a strong specificity towards GTP^39^, possibly still keeping a poor catalytic ability^4^. The reason for this nucleotide specialization lies presumably in how enzymes cope with the intracellular concentrations, or variations, of these two nucleotides and Leipe and colleagues speculated that these GTPases could thus avoid a competition with more abundant, very active ATPases; GTPases could therefore specialize in essential regular functions (e.g. translation or cell signaling) by avoiding too much fluctuation in energy supply^7^. Yet, further evolution steps in the GTPase lineage fashioned some subclasses of proteins that lost their GTP preference due to mutations or complete deletion of the NKxD motif, thus creating ATPases with an otherwise classical GTP-binding fold (e.g. myosin or kinesin). Here, the scenario is very likely reversed with an ABC transporter that switches taste to prefer GTP. We have no evidence for a higher GTP affinity, based on binding experiments with TNP nucleotides and *K*_M_ for NTP hydrolysis. However, the GTPase activity of PatA/PatB is higher than its ATPase activity, both in the absence and the presence of various drugs. In addition, it is possible that these activities are also differently coupled to transport depending on the drug and temperature. At 25 °C, for instance, Hoechst transport can be fueled by ATP, showing that ATPase activity can be well coupled to transport at this temperature. The molecular bases responsible for GTP preference appear subtle because based on PatA/PatB primary sequences (Fig. S12 and Tables S1 and S2) and its modeled 3D structure (Fig. 7), this transporter seemingly complies in every respect to the standard of the ABC transporters. However, we are currently constructing and analyzing BmrC/BmrD and PatA/PatB chimera to pinpoint the molecular determinants for GTP preference in PatA/PatB.

Why would an ABC transporter shift its preference towards GTP over ATP? ATP has been found to be the most abundant nucleotide in glucose-fed exponentially grown *E*. *coli* (∼10 mM), slightly above the UTP concentration, but GTP was quite abundant too (∼5 mM)^40^. Yet, in a whole *E*. *coli* population, individual bacteria can have very different levels of intracellular ATP concentrations, ranging from less than 0.5 mM to more than 5 mM^41^. This result stresses how variable the intracellular ATP concentration can be and impart that the mean value should be considered with caution, as a large dispersion exists within each individual even if they originate from a unique cell. Although the same conclusion might also be true for the intracellular GTP concentrations, it is possible that GTP can readily be found in excess over ATP concentration in some physiological conditions, and among a single bacterium, regardless of the average values found for the whole population. In addition, *S*. *pneumoniae* is a facultative anaerobe incapable of respiratory metabolism^42^. It therefore gets its energy exclusively through fermentation of carbohydrates that are oxidized to pyruvate during glycolysis, as is found in most if not all streptococcal species^43^. Consequently, the replenishment in ATP might not be as efficient as in higher eukaryotes, and for instance it has been estimated that a human body consumes every day the equivalent of its weight in ATP with the regeneration of this nucleotide accomplished by the mitochondria^44^. It remains to be investigated whether the preference for GTP is a conserved feature in other ABC transporters from *S*. *pneumoniae*. Yet, it is tempting to speculate that the physiological xenobiotic substrate(s) of this transporter found in *S*. *pneumoniae* habitat, which is (are) still unknown, could also increase the pool of intracellular GTP, as found here with norfloxacin, ciprofloxacin and acriflavin. Furthermore, since fluoroquinolones promote DNA damage, a fraction of the cellular ATP may be consumed by DNA repair enzymes^45,46^. Throughout evolution of the pneumococcus, one of these constraints has favored the advent of GTP preference over ATP to energize PatA/PatB.

Is PatA/PatB the only transporter that prefers GTP? As mentioned above, ATP is used exclusively by primary transporters but one noticeable exception is found with the iron bacterial transporter, FeoB, which imports ferrous iron under anaerobic condition. Nevertheless, most of the transporters in this ‘exotic’ family contain a typical G-domain with all the conserved motifs found in classical GTPases^7^. Yet, it is unclear whether they really function as primary transporters or as channels gated by the GTPase activity of FeoB^47^. In the ABC transporter family, several hints indicated that some transporters could use other nucleotide, besides ATP, as energy source. Hence, CvaB, which is involved in the export of colicin V in *E*. *coli*, has been shown to process this peptide slightly better with GTP than with ATP in an *in vitro* assay^21^. In addition, while the isolated NBD of CvaB shows similar GTPase and ATPase activities at 37 °C, lowering the temperature to 10 °C revealed its preference for GTP^48,49^. In yeast, it has been proposed that the multidrug Pdr5p expands its substrate specificity by using GTP as an alternative source of energy^18^. Although intriguing, the physiological relevance of these results was not firmly established. Here, we show that PatA/PatB is a deviant ABC transporter that has evolved to favor GTP as a cellular fuel and this transporter might represent the first example of a new subclass of ABC transporters that we propose to dub GTP-Binding Cassette transporters. Our results widen our vision of the ABC transporters superfamily, one of the oldest protein superfamilies^50^, and of the primary transporters in general, and urge to thoroughly study the nucleotide need of each transporter.

## Materials and Methods

### Bacteria and growth conditions

*E*. *coli* C41(DE3) or BL21(DE3) strains were used for PatA/PatB or MSP1E3D1 overexpression, respectively. The bacteria were grown in Terrific Broth (TB) medium at 37 °C, under 150 rpm. *S*. *pneumoniae* R6 strains wild type and *ΔpatA/patB* mutant^24^ were grown in Bacto Todd Hewitt (TH) broth at 37 °C under 5% CO_2_ in air.

### Proteins overexpression and preparation of inside-out membrane vesicles

Recombinant PatA/PatB was produced in *E*. *coli* C41(DE3) strain as described previously^24^. Freshly-transformed bacteria were grown at 37 °C until OD_600_ reached 2–2.5. Protein expression was induced with 0.7 mM (IPTG) for 13–15 h at 25 °C. Bacteria were collected by a 4,000 g centrifugation for 15 min at 4 °C and re-suspended in 45 mL of buffer A per culture liter (50 mM Tris-HCl pH 8, 5 mM MgCl_2_, 1 mM DTT, 5 μg/mL DNAse, 5 μg/mL RNAse, one tablet of protease inhibitor cocktail “complete EDTA-free”). Bacteria were lysed by three successive passages through a microfluidizer at 18,000 psi. All subsequent steps were carried out at 4 °C. Unbroken cells and cell debris were removed by 15,000 g centrifugation for 30 min. Membrane vesicles were collected by centrifugation for 1 h at 150,000 g and re-suspended in 50 mM Tris-HCl pH 8, 1.5 mM EDTA and centrifuged again for 1 h at the same speed. Membranes were finally re-suspended in 20 mM Tris-HCl pH 8.0, 1 mM EDTA and 300 mM sucrose, frozen as small aliquots in liquid nitrogen and stored at −80 °C. Membranes overexpressing BmrC/BmrD were prepared as described earlier^27^. Membrane concentration was determined by a modified Lowry protein assay from Pierce.

### Transport assays

For drug transport assays, fluorescence change of Hoechst 33342 (2′-(4-ethoxyphenyl)-5-(4-methyl-1-piperazinyl)-2,5′-bis-1H-benzimidazole) and doxorubicin were monitored with a Photon Technology International Quanta Master I fluorimeter at the temperature specified in the figure legends. For Hoechst 33342 transport assays, the excitation and emission wavelengths were set at 355 and 457 nm, respectively. For doxorubicin experiments, the excitation and emission wavelengths were set at 480 and 590 nm, respectively. Briefly, inside-out membrane vesicles (50 µg of total proteins when using membranes expressing PatA/PatB, except when stated otherwise, and 200 µg of total proteins when using membranes expressing BmrC/BmrD) were added to a 3 mL cuvette containing 1 mL of 50 mM Hepes-KOH pH 8.0, 2 mM MgCl_2_, 8.5 mM NaCl, 60 µg of pyruvate kinase, and 4 mM phosphoenolpyruvate. After incubation for 1 min, 1 µM of Hoechst 33342 or 2 µM of doxorubicin were added, and its fluorescence was recorded for ~1 min. Nucleotides (2 mM, unless stated otherwise) were then added, and the fluorescence intensity was monitored for several min.

### Purification of PatA/PatB heterodimers

Membranes were solubilized for 2.5–3 h on ice in solubilization buffer (50 mM Tris-HCl pH 8, 15% glycerol, 100 mM NaCl, 10 mM imidazole and 5 mM β-mercaptoethanol) supplemented with 1% lauryl maltose neopentyl glycol (LMNG) at a protein concentration of 2 mg/mL. Solubilized membranes were centrifuged for 1 h at 150,000 g. The supernatant was incubated for 1–2.5 h with a Ni-NTA resin. Resin was poured into a column and extensively washed with solubilization buffer containing 20 mM imidazole and 0.02% LMNG. Elution was performed with the same buffer containing 250 mM imidazole. Eluted protein was dialyzed overnight in 50 mM Tris-HCl pH 8, 10% glycerol, 100 mM NaCl, 5 mM β-mercaptoethanol and 0.005% LMNG. Samples were then submitted to gel filtration on a HiLoad^TM^ 16/60 Superdex^TM^ 200 prep grade or Superdex^TM^ 200 10/300 GL column using the same buffer composition. PatA/PatB was concentrated with Amicon Ultra centrifugal filters (100 kDa cut-off) and frozen in liquid nitrogen before storage at −80 °C. Protein concentration was determined by a Bradford protein assay.

### Reconstitution of PatA/PatB into proteoliposomes

It was performed as described before for the ABC transporter BmrA^36^.

### Purification of MSP1E3D1

For His-tagged membrane scaffold protein MSP1E3D1 production, protein overexpression was induced with 1 mM IPTG for 3 h at 37 °C. Bacteria were pelleted by low speed centrifugation and re-suspended in lysis buffer (40 mM Tris pH 7.4, 1 mM PMSF, 1% Triton X-100 and 30 mg/mL DNAse). Bacterial lysis was performed by sonication on ice for 25 min (150 ultrasound cycles of 2 sec on and 8 sec off). Soluble material was recovered by centrifugation at 30,000 g for 1 h and loaded onto a Ni-NTA-agarose column. Resin was washed successively with 50 mL of buffer 1 (40 mM Tris-HCl pH 8, 0.3 M NaCl, 1% Triton X-100), 25 mL of buffer 2 (40 mM Tris-HCl pH 8, 0.3 M NaCl, 50 mM sodium cholate, 20 mM imidazole) and 35 mL of buffer 3 (40 mM Tris-HCl pH 8, 0.3 M NaCl, 50 mM imidazole). Elution was carried out with 400 mM imidazole. Purified His-tagged MSP1E3D1 was dialyzed in 50 mM Tris-HCl pH 8 and 100 mM NaCl for 2 h. After cleavage of the His6 tag with the TEV protease, MSP1E3D1 was dialyzed overnight against a 20 mM Tris-HCl pH 7.4, 100 mM NaCl, 0.5 mM EDTA buffer, and was concentrated on Centricon Ultra-15 (10 kDa molecular weight cutoff). Protein concentration was determined by measuring absorbance at 280 nm.

### *S*. *pneumoniae* lipid extraction

*S*. *pneumoniae* R6 strain was grown in TH medium. After centrifugation of the culture, the pellet (3.5 g) was suspended into 3.5 mL of Tris-HCl 100 mM pH 7.0. Peptidoglycan was digested by addition of lysozyme (2 mg/mL) for 1 h on ice. Bacterial suspension was subjected to biphasic lipid extraction. A 2/1/1 (v/v/v) solution of methanol, chloroform and water was added and the mixture was vigorously shaken for 5 min, then incubated for 15 min at room temperature. Then 150 mL of chloroform/water 1/1 (v/v) supplemented with 1.3 mL of 6 M HCl were added to obtain a final ratio of 1/1/1 (v/v/v) methanol/chloroform/water and incubated for 1 h at room temperature and then overnight at 4 °C. The lower phase containing lipids was removed. The upper and intermediate phases were re-extracted with chloroform by mixing and incubation as described above. The chloroform phases from the repetitive extractions were collected and supplemented with 100 mM NaCl, 500 mM Tris-HCl pH 8.2, 100 mM EDTA. The mixture was shaken and the chloroform containing phase was collected and dried in a glass balloon using a gentle stream of nitrogen under rotation until total evaporation of the solvent to recover the *S*. *pneumoniae* lipid extract.

### Incorporation of PatA/PatB into nanodiscs

Dried lipids from *E*. *coli* (Avanti Polar Lipids, Inc.) or from *S*. *pneumoniae* were solubilized in a 20 mM Tris-HCl pH 7.4 buffer containing 4.9% LMNG by incubation at 37 °C under 150 rpm. When solubilized, lipids were mixed with MSP1E3D1 and PatA/PatB in a ratio of 2/2/1 (w/w/w) and incubated for 1 h at room temperature before adding 650 mg/mL of Biobeads (BioRad) allowing detergent absorption and nanodiscs formation. After filtration on a 0.45 µm PVDF membrane filter, the mixture was submitted to an affinity chromatography using Ni-NTA resin gel followed by a gel filtration (HiLoadTM 16/60 SuperdexTM 200 prep grade column) in a buffer containing 20 mM Tris-HCl pH 7.4, 150 mM NaCl and 10% glycerol. In-gel quantification of incorporated recombinant proteins in nanodiscs was performed on 11% Tris-Tricine gel using a range of pure recombinant proteins of known concentration as a standard. Protein concentration was determined using the Image J software.

### Determination of ATPase and GTPase activities

ATPase and GTPase activities were determined by a coupled enzymatic assay^51^ that regenerates ATP and GTP with the concomitant NADH oxidation. Enzymatic activities were assessed through the use of a spectrophotometer (UVmc^2^ Safas or V-630Bio Jacso). For proteoliposomes, 4 µg of PatA/PatB were added to 50 mM Hepes/KOH pH 8, 10 mM MgCl_2_, 60 µg/mL pyruvate kinase, 32 µg/mL lactate dehydrogenase, 4 mM phosphoenolpyruvate and 0.3 mM NADH. For nanodiscs experiments, 4 µg of PatA/PatB incorporated into nanodiscs were added to a 20 mM Tris-HCl pH 7.4, 150 mM NaCl and 10% glycerol buffer supplemented with 2 mM MgCl_2_, 40 µg/mL lactate dehydrogenase, 80 µg/mL pyruvate kinase, 8 mM phosphoenolpyruvate and 0.8 mM NADH. Absorbance at 340 nm was recorded at various temperatures upon addition of nucleotides, ATP or GTP, at the concentration indicated in the figure legends. When specified, 4 mM of orthovanadate was added to the mixture. When GTP concentrations were ≥5 mM, NADH concentration was doubled due to the significant amount of contaminating GDP that consumed most of the initial NADH concentration through GTP regeneration.

### Binding of fluorescent nucleotide analogues

Binding of 2′,3′-O-(2,4,6-trinitroPhenyl)-ATP (TNP-ATP) and 2′,3′-O-(2,4,6-trinitroPhenyl)-GTP (TNP-GTP) on purified PatA/PatB was monitored by extrinsic fluorescence at 25 °C. Two 1 ml cuvettes were prepared: one with protein dialysis buffer alone (50 mM Tris-HCl pH 8, 15% glycerol, 100 mM NaCl, 1 mM EDTA, 0.02% LMNG), and one which contains in addition PatA/PatB at 0.75 µM. Changes in the fluorescence intensity as a function of TNP-nucleotide concentration were recorded with an excitation wavelength of 408 nm and emission spectra monitored from 500–600 nm using a Photon Technology International Quanta Master fluorimeter. Successive additions of TNP-nucleotides (200 µM each) were performed to each cuvette. To analyze the results, fluorescence from the buffer was subtracted from the fluorescence of the protein sample at each TNP concentration. Data were fitted according to the equation F = Fmin + {(Fmax-Fmin) [(Et + L + Kd)-((Et + L + Kd)2–4EtL)1/2]}/2Et^52^ using Grafit software, where Fmax is the fluorescence intensity at saturating concentration of TNP-nucleotide, Fmin the fluorescence intensity at the start of the titration, Et is the total concentration of PatA/PatB and L is the TNP-nucleotide concentration.

### Extraction of intracellular nucleotides from *S*. *pneumoniae* R6 strain

*S*. *pneumonia* R6 strain was grown in TH medium. Cultures were incubated at 30 °C or 37 °C until the absorbance at 600 nm reached 0.25, 0.5 or 1. Metabolite extraction was then performed as we described before^53^. Briefly, the appropriate volume of culture containing 2.5 10^9^ bacteria were immediately filtered onto a Supor450 membrane discs filter 0.45 µm and washed with 5 mL of ice cold NaCl 0.6%. Filters were quickly transferred into cold 60% ethanol (−20 °C) and were flash-frozen in liquid nitrogen. Cell lysis was induced by vortexing the samples with glass bead for 4 min. Cell debris were pelleted by centrifugation for 5 min. Supernatants were dried under speed vacuum until total evaporation. An identical procedure was applied to cultures exposed to norfloxacin (16 µg/mL), acriflavin (8 µg/mL), berberin (32 µg/mL) or ciprofloxacin (4 µg/mL) for 5 to 60 min.

### Determination of nucleotide concentration by mass spectrometry

Dried samples were solubilized by 80 µL of a 60/40 (v/v) solution of acetonitrile/10 mM ammonium carbonate buffer (pH 10.5, adjusted with ammonium hydroxide) containing ^15^N-labeled AMP, ADP, ATP and GTP at 2.0, 2.0, 4.0 and 0.5 µg/mL, respectively. ^15^N-labeled AMP and ATP were provided by Euriso-top (Saint-Aubin, France), while ^15^N-ADP and ^15^N-GTP were from Sigma-Aldrich (Saint Quentin Fallavier, France). LC/MS analyses were performed by coupling an Ultimate 3000 chromatographic system to an Exactive Orbitrap mass spectrometer (Thermo Fisher Scientific, Courtaboeuf, France) essentially as described before^54^. Briefly, the chromatographic separations were performed using a Sequant ZIC-pHILIC (5 μm, 2.1 × 150 mm) column maintained at 15 °C (Merck, Darmstadt, Germany) and operated under gradient elution, as follows. Mobile phases were 10 mM ammonium carbonate (adjusted at pH 10.5 with ammonium hydroxide) (A) and 100% acetonitrile (B), and the flow rate was 200 μL/min. After a 2 min isocratic step at 80% B, nucleotides were eluted using a linear gradient of 80 to 40% B in 10 min. The Exactive mass spectrometer was operated in the negative ion mode using a capillary voltage and a capillary temperature set at −3 kV and 280 °C, respectively. The sheath gas pressure and the auxiliary gas pressure (nitrogen) were fixed at 60 and 10 arbitrary units, respectively. The detection was performed from *m/z* 75 to 1000 using a resolution set at 50,000 at *m/z* 200 (full width at half maximum).

Nucleotides were quantified by the isotope dilution method using their ^15^N-labeled homologues as described before^53^.

### Construction of 3D models of PatA/PatB in two conformational states

3D models of heterodimer PatA/PatB were built using Modeller9v14^55,56^, using the crystal structures of *Thermotoga maritima* heterodimer TM287/TM288 (PDB 3QF4) and *Staphylococcus aureus* homodimer Sav1866 (PDB 2ONJ) as templates, for rebuilding inward-facing and outward-facing conformations respectively. The selection of the template structures in PDB for PatA/PatB model rebuilding followed different criteria: the availability of ABC heterodimer or homodimer ABC transporter structures with the best possible similarity, the highest alignment length, the atomic resolution, and the presence of bound nucleotide co-crystallized in NBD domains. An additional criterion that had to be taken into account was the conformational state, since ABC transporters have been crystallized in various states presumed to reflect distinct steps in the catalytic cycle, between two opposite configurations referred to as open inward-facing and outward-facing conformations. With the above criteria, only two potential templates in the inward-facing conformation were suitable, the heterodimers TM287/TM288 and TmrA/TmrB (from *Thermus thermophilus*, PDB 5MKK, recently released). These two structures were resolved at high and comparable atomic resolution (2.9 and 2.7 Å respectively), but the sequence similarities found between PatA/B and TM287/288 or TmrA/B (see Tables S1 and S2) favored a mono-template rebuilding strategy based on TM287/TM288 structure with a favorable average identity of 38–39%. For rebuilding in outward-facing conformation, only two templates in PDB could be considered, the homodimers Sav1866 (from *Staphylococcus aureus*, PDB 2ONJ) and MsbA (from *Salmonella Typhimurium*, PDB 3B60). PatA and PatB exhibited similar sequence identities with Sav1866 and MsbA (about 30% for each Pat protein with Sav1886, and 27 and 31% for PatA and PatB, respectively, with MsbA), but due to higher atomic resolution (3.4 Å versus 3.7 Å), the Sav1866 structure 2ONJ was proposed to be the unique template for homology modeling of PatA/PatB in the outward-facing state. Due to sequence length of templates, PatA/PatB models based on TM287/288 were full-length rebuilt structures (PatA/1-564, PatB/1-603), whereas models based on half transporter Sav1866 included full-length PatA (1–564) and N-edge truncated PatB (22–603).

For each monotemplate-based structure, runs of 100 models were performed with a further loop refinement protocol, and the generated models sorted by the MODELLER objective function were evaluated by their DOPE (Discrete Optimized Protein Energy) and GA341 scores calculated by Modeller. Best models (corresponding to the lowest DOPE scores) issued from each run were submitted for model quality assessment to further evaluation scoring on the QMEAN server^57^ using QMEAN scoring function^58^ and the new QMEANbrane score designed for membrane proteins^59^. The final models displayed good pseudo-energy outputs of membrane locating algorithm, placing them within the expected range of transmembrane structures, and displayed good QMEAN4 and QMEAN6 values, as compared to the QMEAN scores calculated on their respective templates. As a result, the selected PatA/PatB models based on TM287/288 and Sav1866 showed QMEAN scores equal to 0.63 and 0.59 respectively, to be compared to 0.68 and 0.60 values of individual PDB templates respectively.

### Data availability

The datasets generated during and/or analysed during the current study are available from the corresponding author on reasonable request.

## Electronic supplementary material

Supplementary information
