# Supplementary material for: A multidrug ABC transporter with a taste for GTP
Source: Sci Rep. 2018 Feb 2;8:2309. doi: 10.1038/s41598-018-20558-z (PMC5797166; doi:10.1038/s41598-018-20558-z)
Supplement: Supplementary file 1 — Supplementary information [file 41598_2018_20558_MOESM1_ESM.doc]

**SUPPLEMENTARY INFORMATION**

**A multidrug ABC transporter with a taste for GTP**

Cédric Orelle^1§^, Claire Durmort^2§*^, Khadija Mathieu^1§^, Benjamin Duchêne^2^, Sandrine Aros^3^, François Fenaille^3^, François André^4^, Christophe Junot^3^, Thierry Vernet^2^ & Jean-Michel Jault^1*^

^1^University of Lyon, CNRS, UMR5086 “Molecular Microbiology and Structural Biochemistry”, IBCP, 7 Passage du Vercors, F-69367 Lyon, France.

^2^Institut de Biologie Structurale (IBS), University Grenoble Alpes, CEA, CNRS, 38044 Grenoble, France.

^3^CEA, Institut Joliot, Service de Pharmacologie et d'Immunoanalyse, UMR 0496, Laboratoire d'Etude du Métabolisme des Médicaments, MetaboHUB-Paris, Université Paris Saclay, F-91191 Gif-sur-Yvette cedex, France,

^4^Laboratoire Stress Oxydant et Détoxication (LSOD), Institute for Integrative Biology of the Cell (I2BC), CEA, CNRS, Univ Paris-Sud, Université Paris-Saclay, F-91198, Gif-sur-Yvette cedex, France

^§^Equivalent contribution of these three authors.

^*^Corresponding authors: [jean-michel.jault@ibcp.fr](mailto:jean-michel.jault@ibcp.fr); [claire.durmort@ibs.fr](mailto:claire.durmort@ibs.fr)

Material requests should be sent to Jean-Michel Jault.

**Table S1. Sequence identity and strong similarity between PatA and related ABC transporters**. The pairwise alignment between two transporters were realized using Jalview 2.10.1 [^1^](#_ENREF_1) and the MUSCLE algorithm ^2^.

| transporters | TM287 | BmrC | EfrC | TmrB | LmrC | Sav1866 |
| --- | --- | --- | --- | --- | --- | --- |
|  | % identity / % strong similarity | | | | | |
| PatA | 39.1 / 23.6 | 30.9 / 25.6 | 57.8 / 22.4 | 30.9 / 24.1 | 57.3 / 19.7 | 30.6 / 29.2 |

**Table S2. Sequence identity and strong similarity between PatB and related ABC transporters**. The pairwise alignment between two transporters were realized using Jalview 2.10.1 [^1^](#_ENREF_1) and the MUSCLE algorithm [^2^](#_ENREF_2).

| transporters | TM288 | BmrD | EfrD | TmrA | LmrD | Sav1866 |
| --- | --- | --- | --- | --- | --- | --- |
|  | % identity / % strong similarity | | | | | |
| PatB | 37.9 / 23.3 | 33.4 / 20.8 | 56.9 / 21.2 | 32.5 / 25.0 | 56.8 / 19.7 | 30.2 / 25.5 |

**Table S3.** **Major phospholipid components in *E. coli* and *S. pneumoniae*.** These values were taken from[^3^](#_ENREF_3). PE, phosphatidylethanolamine; PG, phosphatidylgylcerol; CL, cardiolipin. *The remaining of the phospholipids was essentially diphosphatidylglycerol[^4^](#_ENREF_4).

| Bacterial species | % Total membrane phospholipid | | |
| --- | --- | --- | --- |
|  | PE | PG | CL |
| *E. coli** | 80 | 15 |  |
| *S. pneumoniae* |  | 50 | 50 |

**
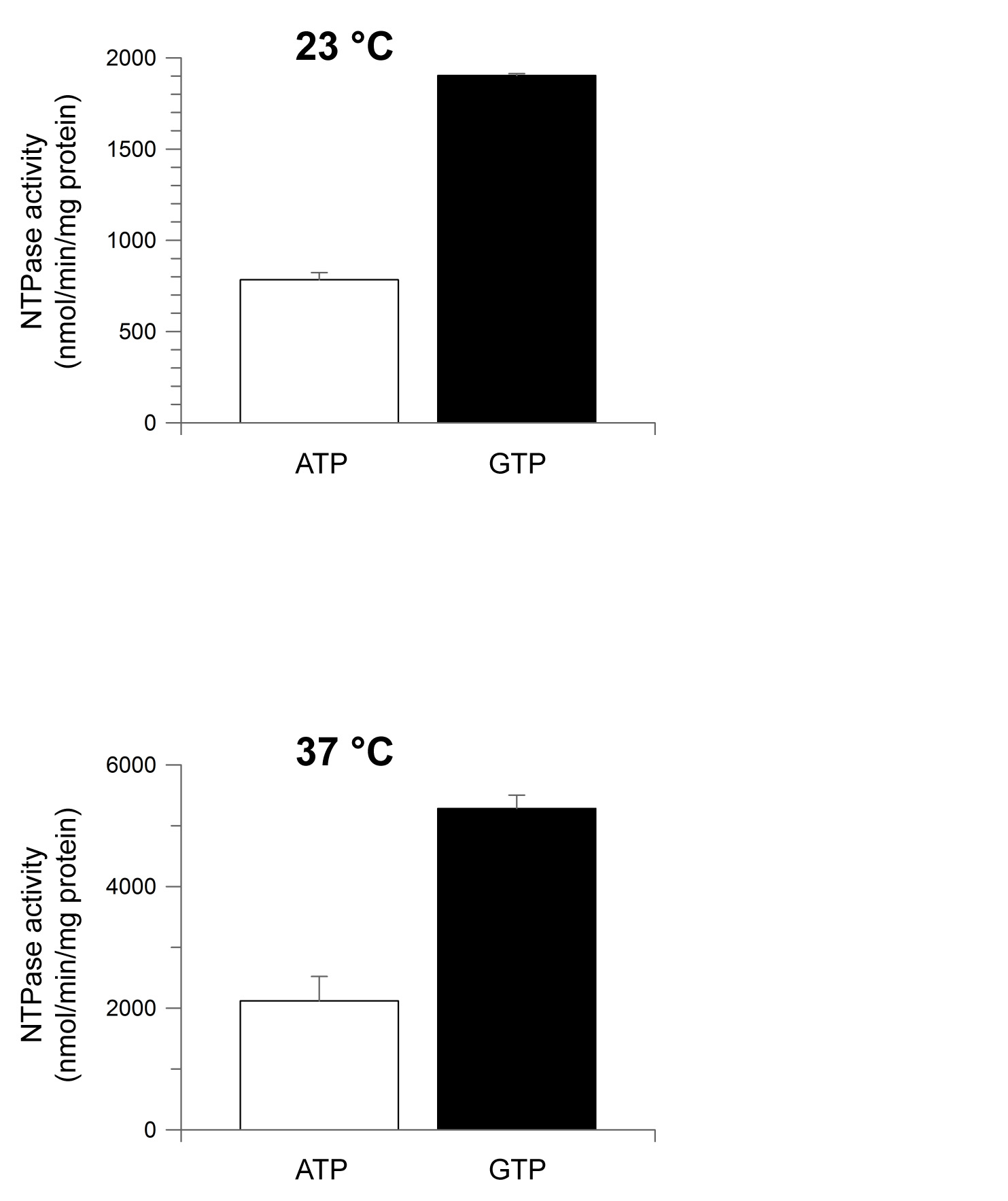
**

**Fig. S1. Hydrolytic activity of purified PatA/PatB.** The ATPase and GTPase activities of PatA/PatB purified in 0.02% LMNG were assayed in the presence of 4 mM nucleotide at either 23 °C or 37 °C. One μg of purified transporter was used in each assay. Triplicates were realized and error bars represent the standard deviation of the mean.

**
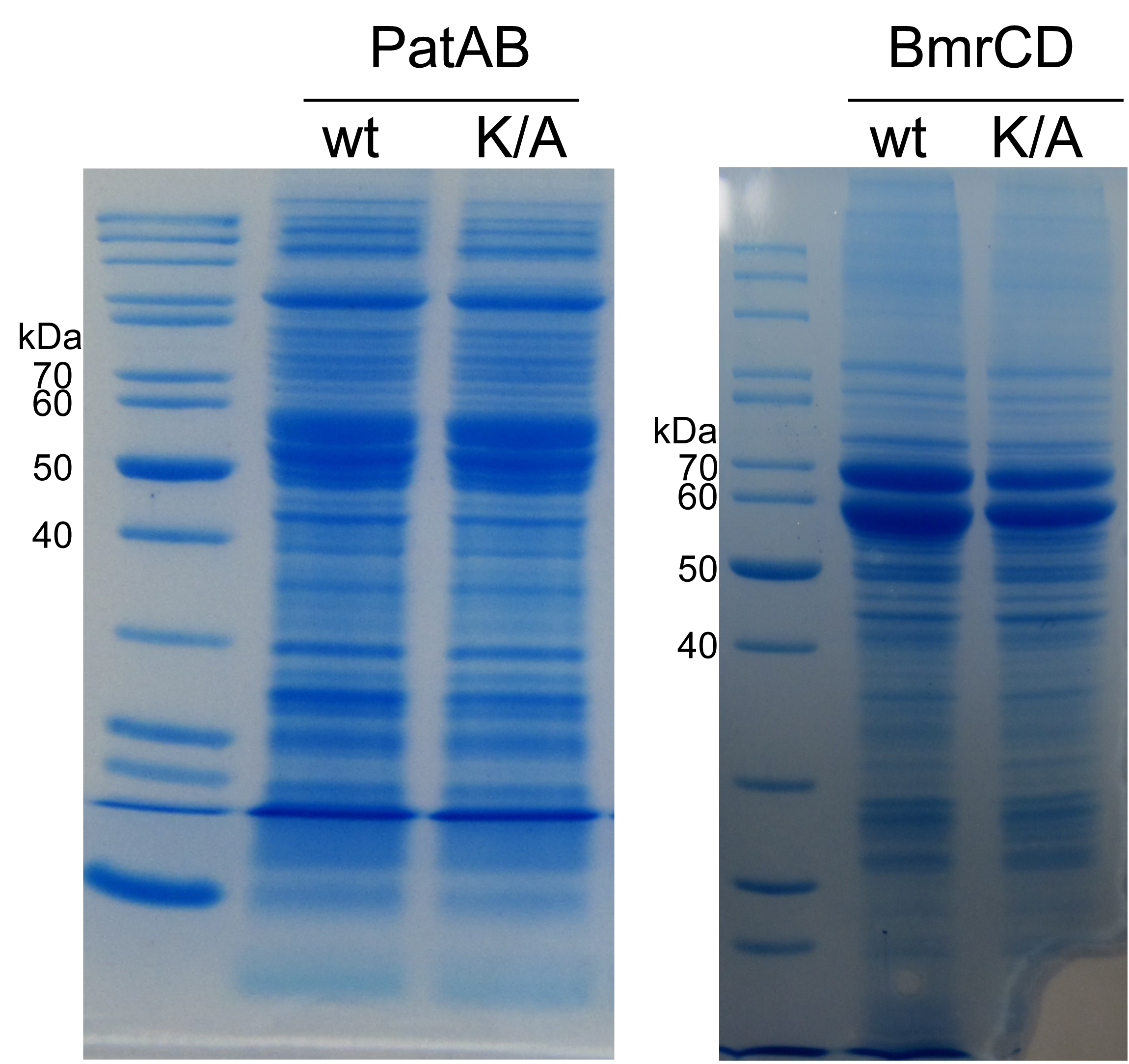
**

**Fig. S2. SDS-PAGE showing the overexpression of PatA/PatB and BmrC/BmrD in *E. coli* membranes.** In the mutants (K/A), the conserved lysine of the Walker-A motif is replaced by an alanine in each subunit.

**
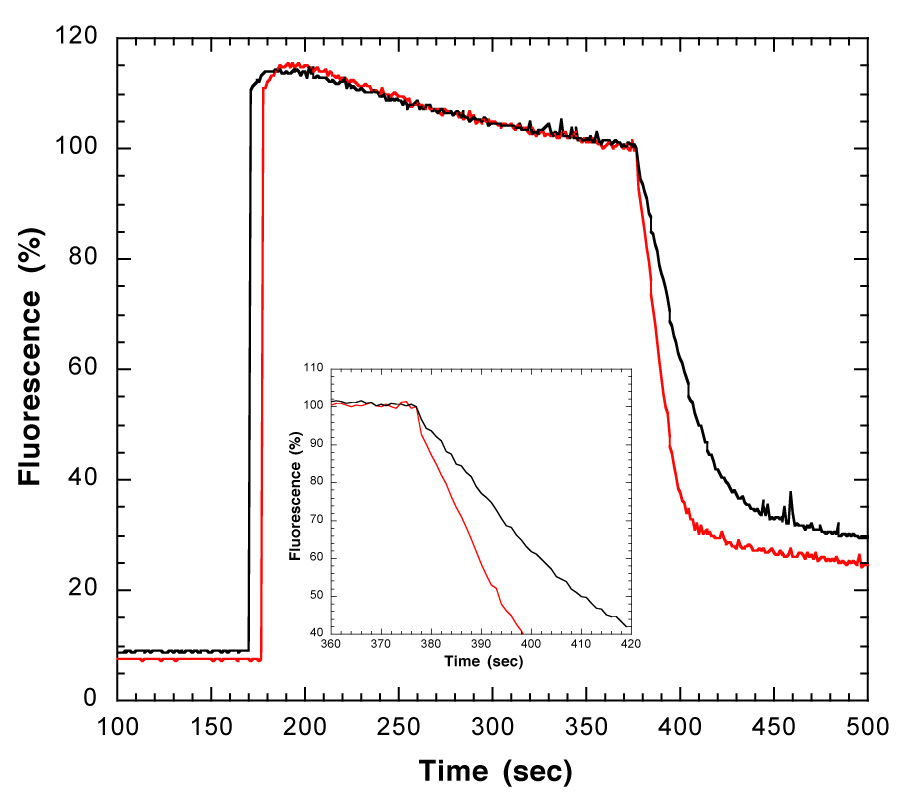
**

**Fig. S3. This figure is similar to Fig1A but with the reference (100% fluorescence) taken at the time where the nucleotide was added to better visualize the difference between the transport rates. The insert shows a close-up view.**

**
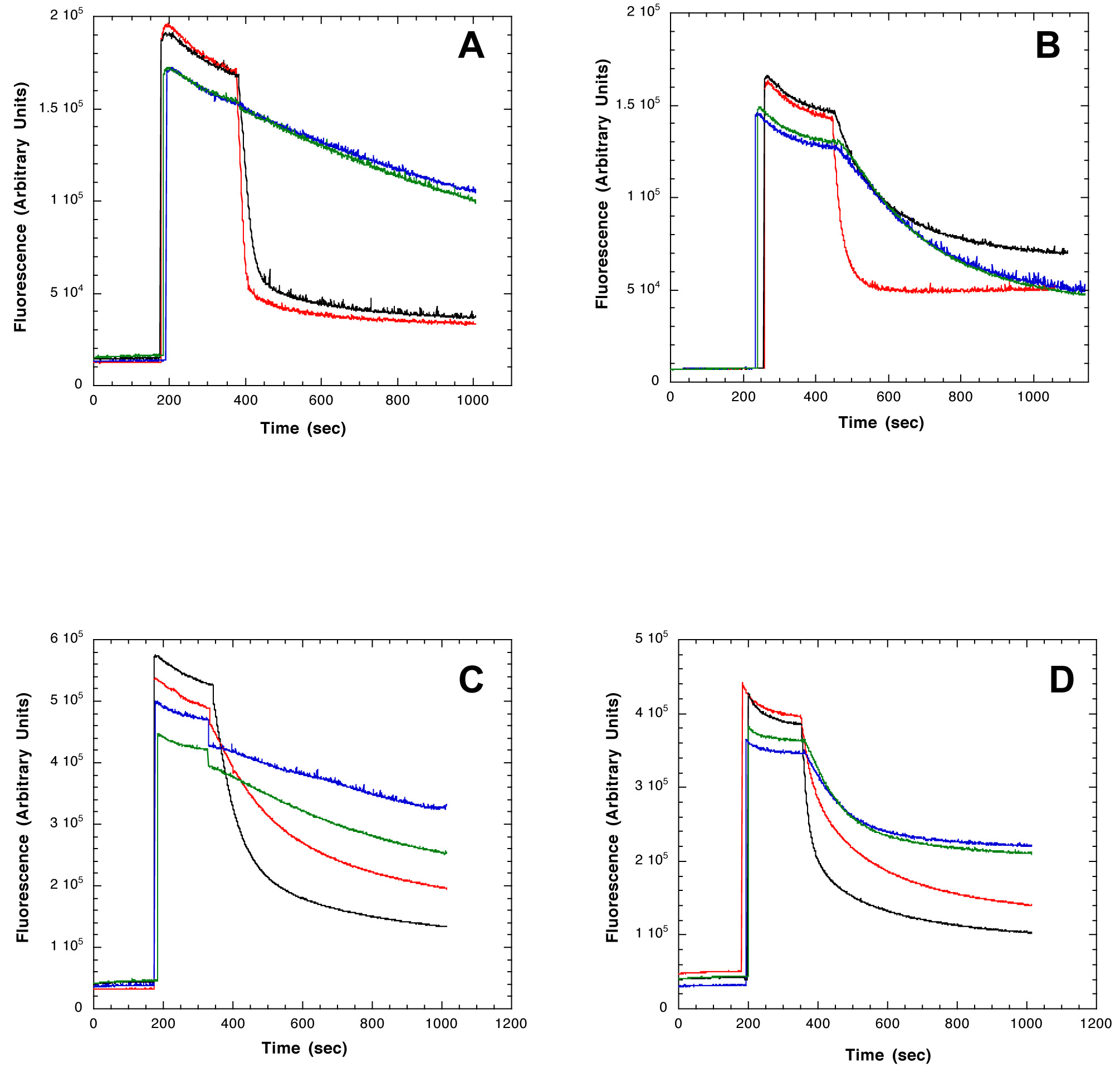
**

**Fig. S4. Hoechst transport of PatA/PatB and BmrC/BmrD.** This figure shows the raw data that were normalized in Fig. 1.

**
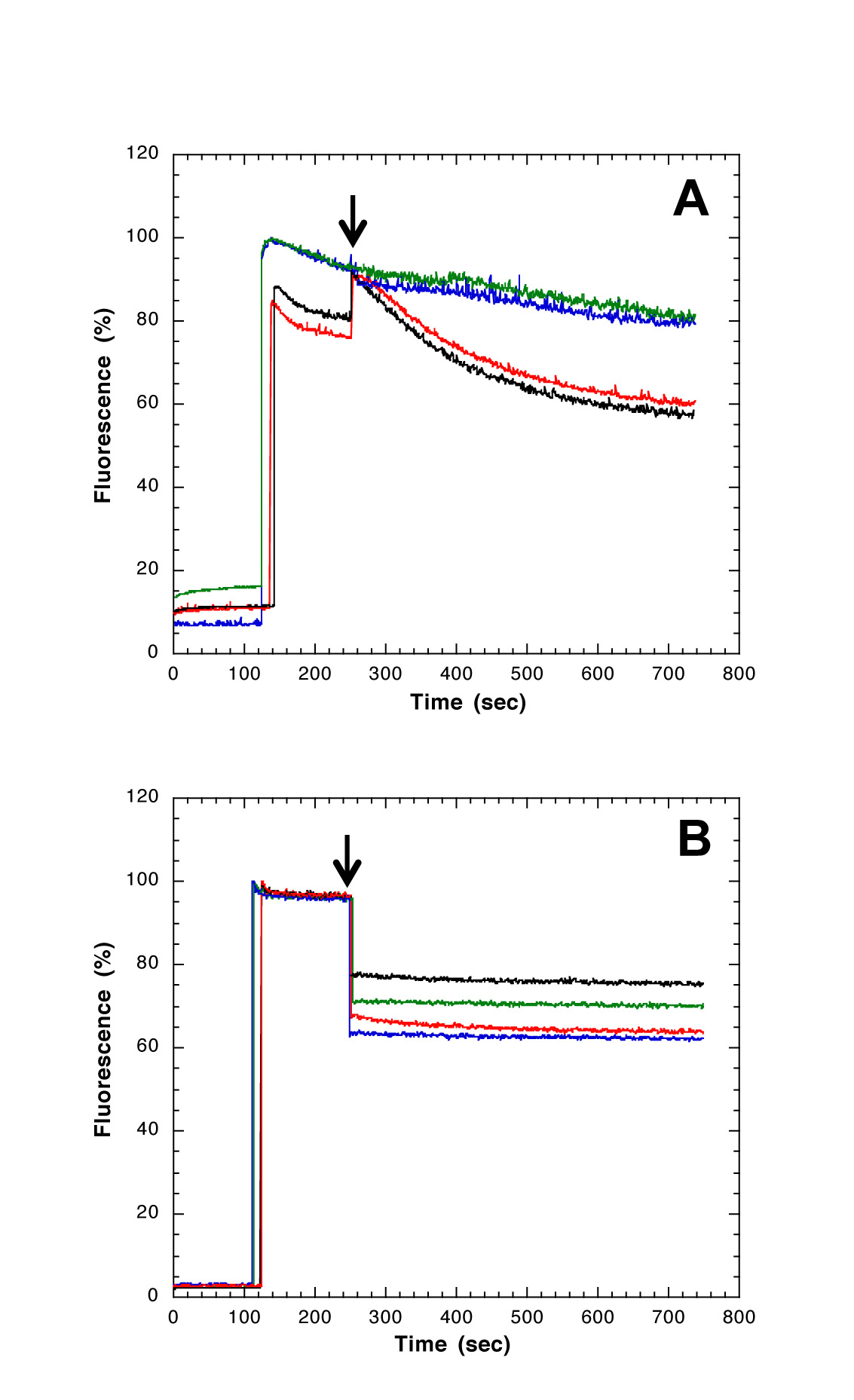
**

**Fig. S5: Drug transport of empty (control) inside-out vesicles.** C41(DE3) cells were transformed by the empty pETDuet-1 vector and the inside-out vesicles were obtained following the same protocol as for those containing overexpressed PatA/PatB. Transport with Hoechst (1 µM, panel ***A***) or with doxorubicin (2 µM, panel ***B***) were performed on the empty inside-out vesicles (50 µg of total membrane protein) at 25 °C (blue and green curves) and 37 °C (red and black curves). The transport was initiated by 2 mM ATP (green and black curves) or 2 mM GTP (blue or red curves).

**
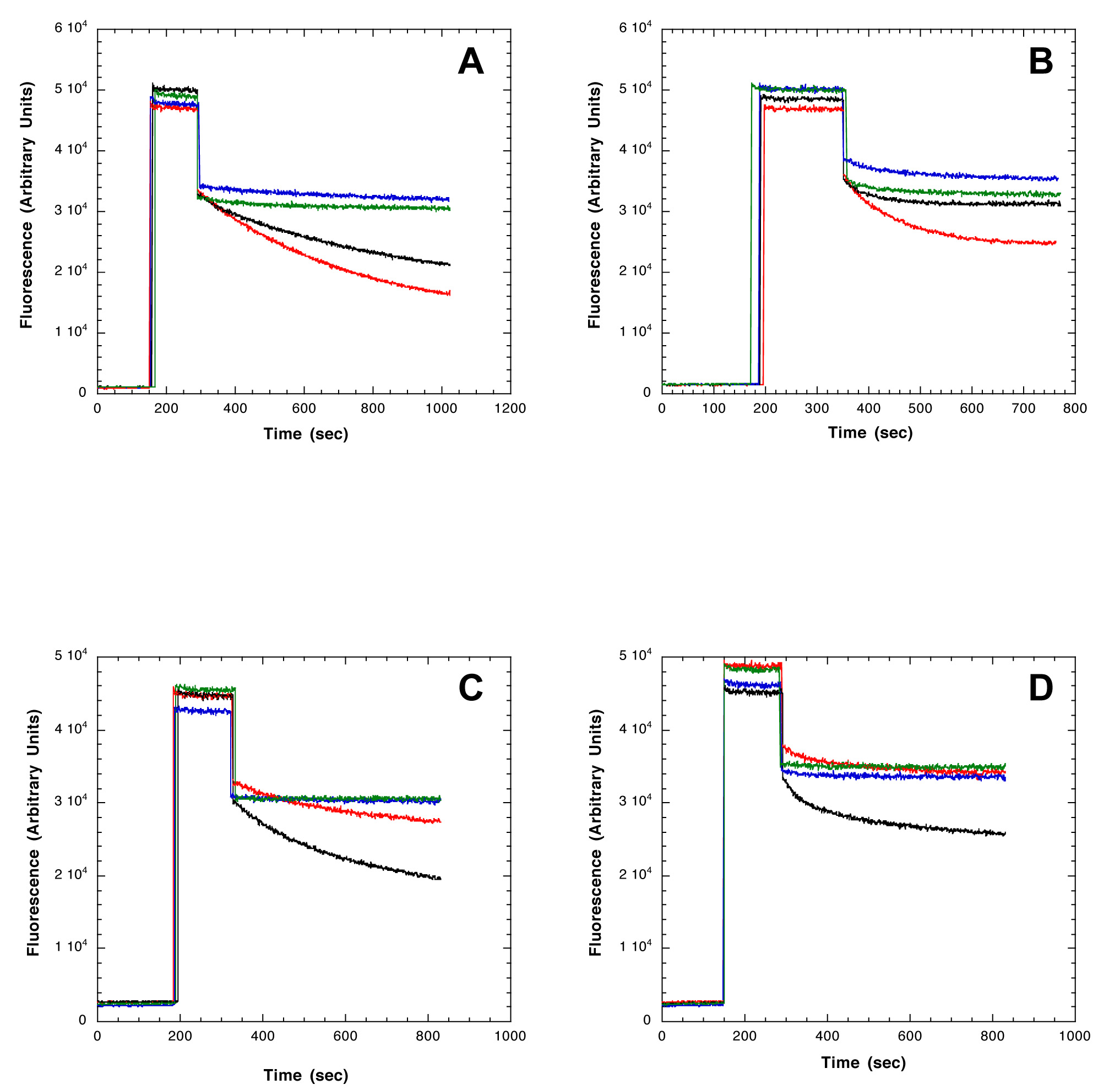
**

**Fig. S6: Doxorubicin transport of PatA/PatB and BmrC/BmrD.** This figure shows the raw data that were normalized in Fig. 1.

**
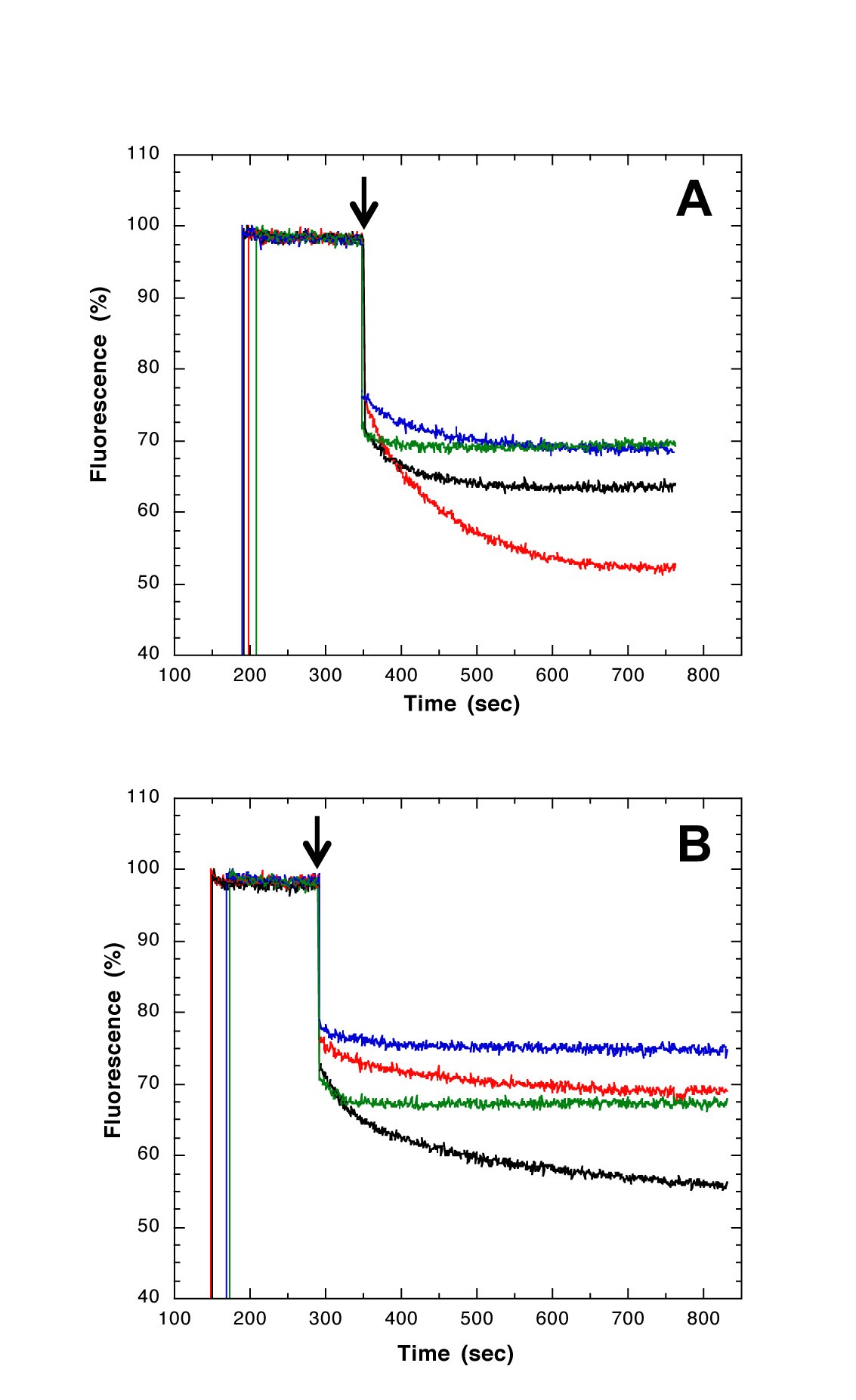
**

**Figure S7. Effect of vanadate on doxorubicin transport of PatA/PatB or BmrC/BmrD.** The experiment was similar to that described in the legend to Fig. 2 and the transport measured at 37 °C was initiated with GTP in the absence (red curve) or in the presence of 2 mM Vi (blue curve), or with ATP in the absence (black curve) or in the presence of 2 mM Vi (green curve). Transports were performed with inside-out vesicles containing overexpressed PatA/PatB (panel ***A***) or overexpressed BmrC/BmrD (panel ***B***).

**
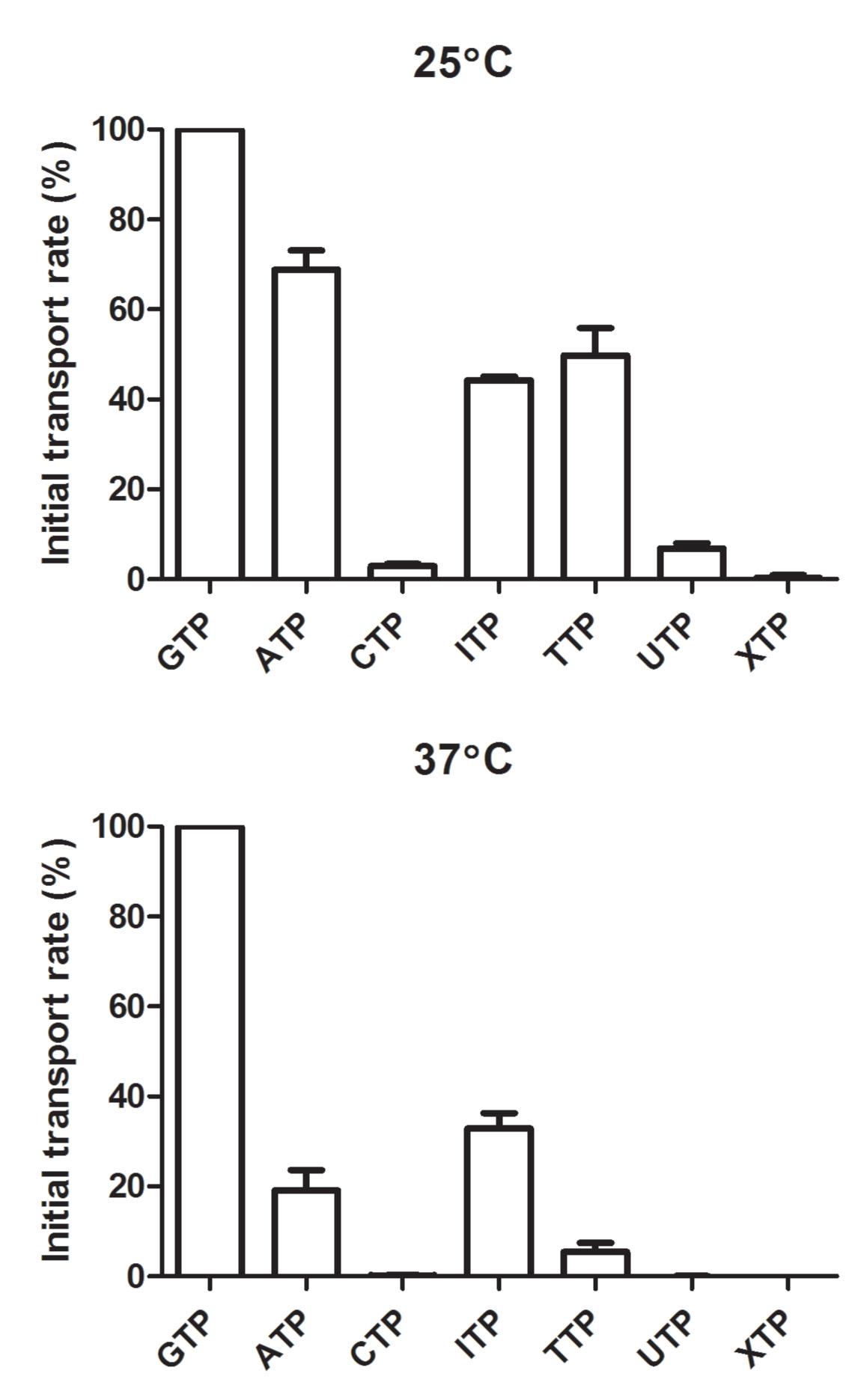
**

**Fig. S8. Effect of different nucleotides to energize Hoechst transport by PatA/PatB**. Hoechst 33342 (1 µM) transport by PatA/PatB was energized with 2 mM of GTP, ATP, CTP, ITP, TTP, UTP or XTP at 25 °C or 37 °C and the initial rates of transport were calculated from the initial slope after nucleotide addition. Transport rates were corrected from the basal transport rates displayed by the membranes containing the inactive PatA/PatB Walker A double mutant. Error bars represent the standard deviation of the mean (n = 3).

**
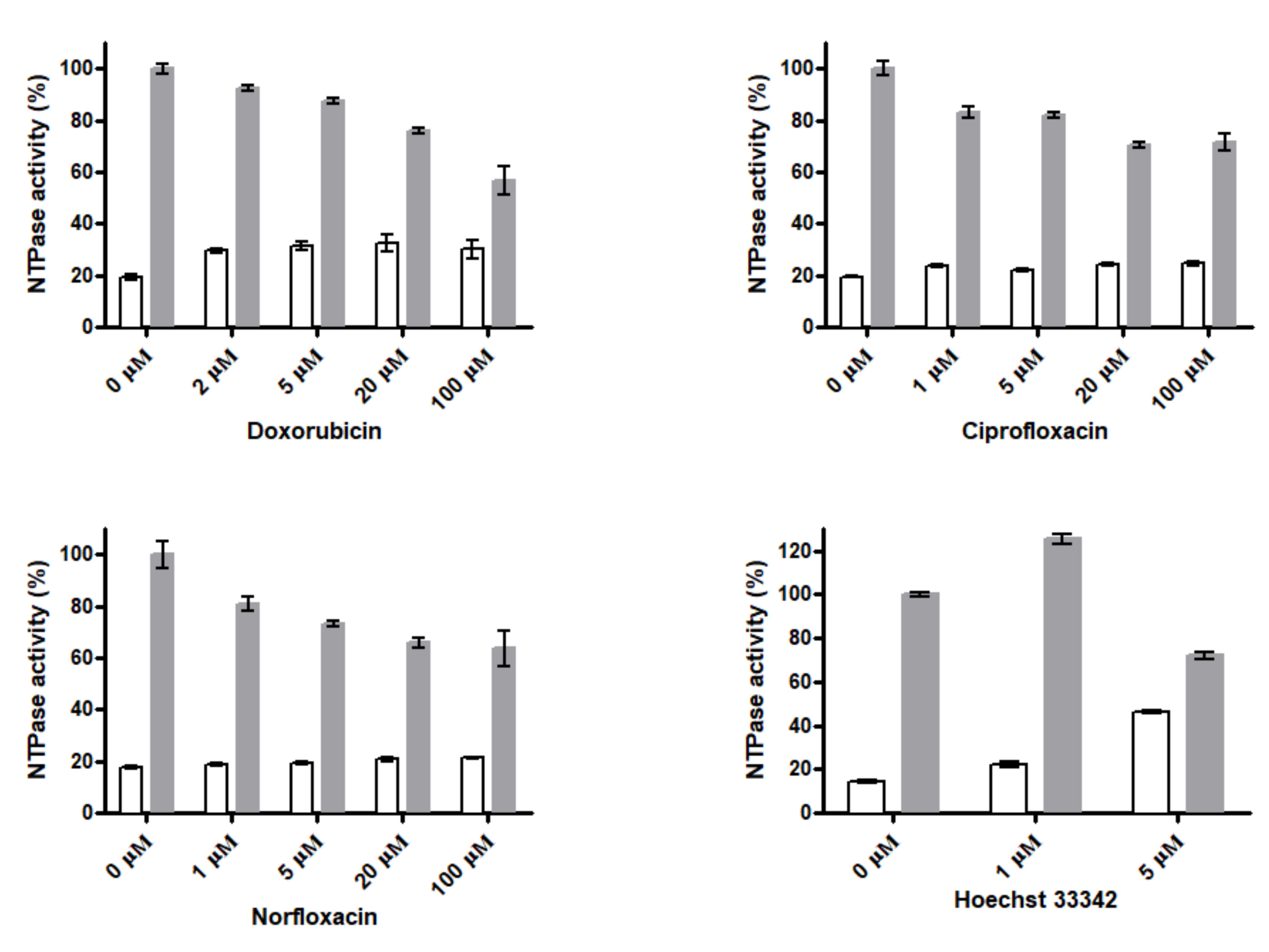
**

**Fig. S9. NTPase activities of PatA/PatB in the presence of various drugs.** ATPase and GTPase activities are displayed as white and grey bars, respectively. Experiments are the average of 3 measurements. Hoechst 33342 was not tested above 5 μM due to interference with the NADH coupled assay at higher concentrations. Note that transport experiments (Fig. 1 and 2) were performed with 1 μM Hoechst 33342 and 2 μM doxorubicin, respectively.

**
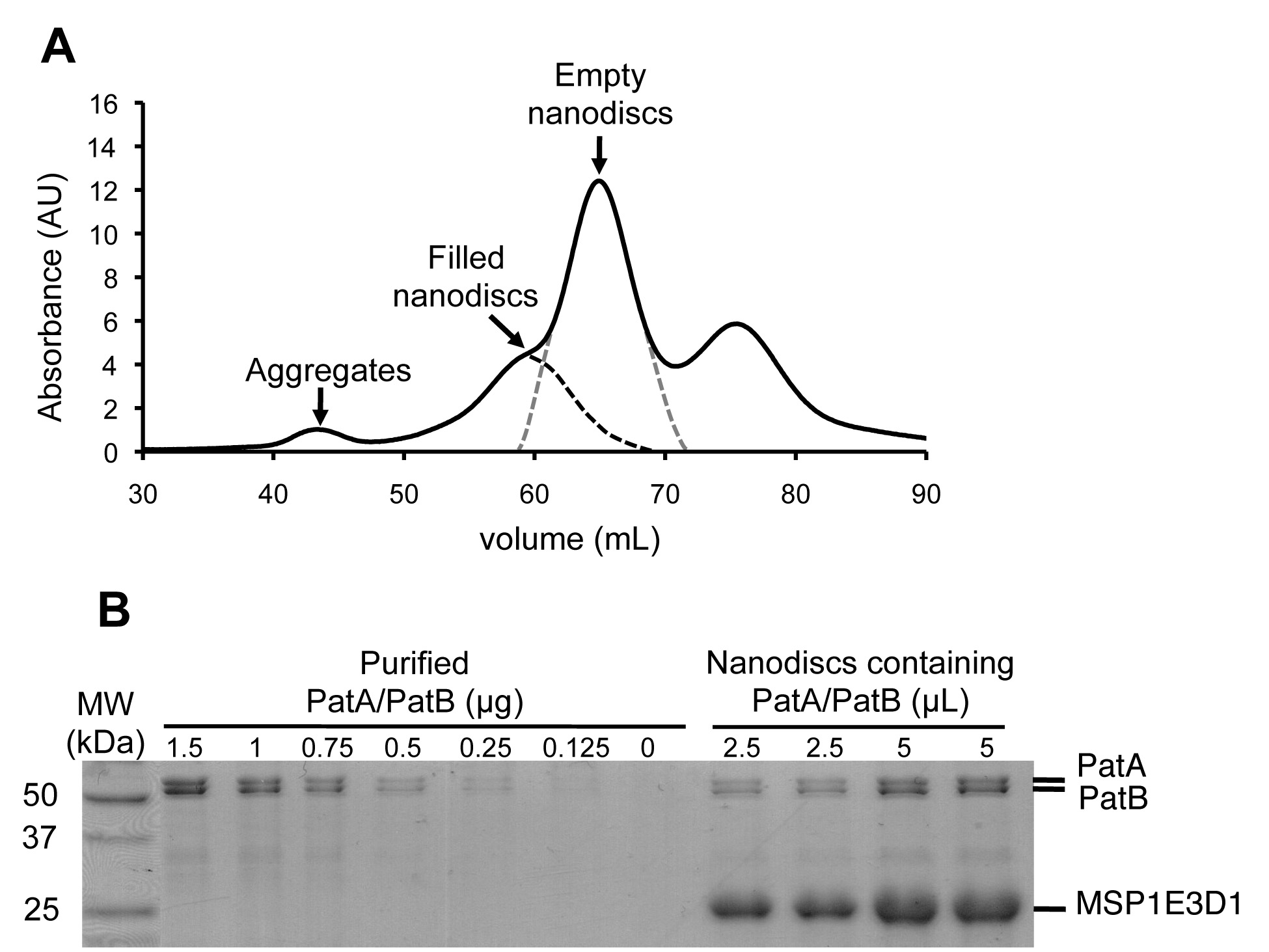
**

**Fig. S10.** **Purification of PatA/PatB reconstituted into nanodiscs.** ***A***, elution profil of a size exclusion chromatography after reconstitution of PatA/PatB into nanodiscs. The elution peaks of aggregates, filled nanodiscs and empty nanodiscs are indicated. ***B***, the amount of PatA/PatB incorporated into nanodiscs was quantified by Tris-Tricine gel electrophoresis (11%). A calibration curve was prepared from detergent-purified PatA/PatB of known concentration. Purified nanodiscs containing PatA/PatB (2.5 µL and 5 µL) were loaded on the gel and intensities of the bands were determined using the Image J software and compared to the calibration curve to estimate the PatA/PatB concentration.


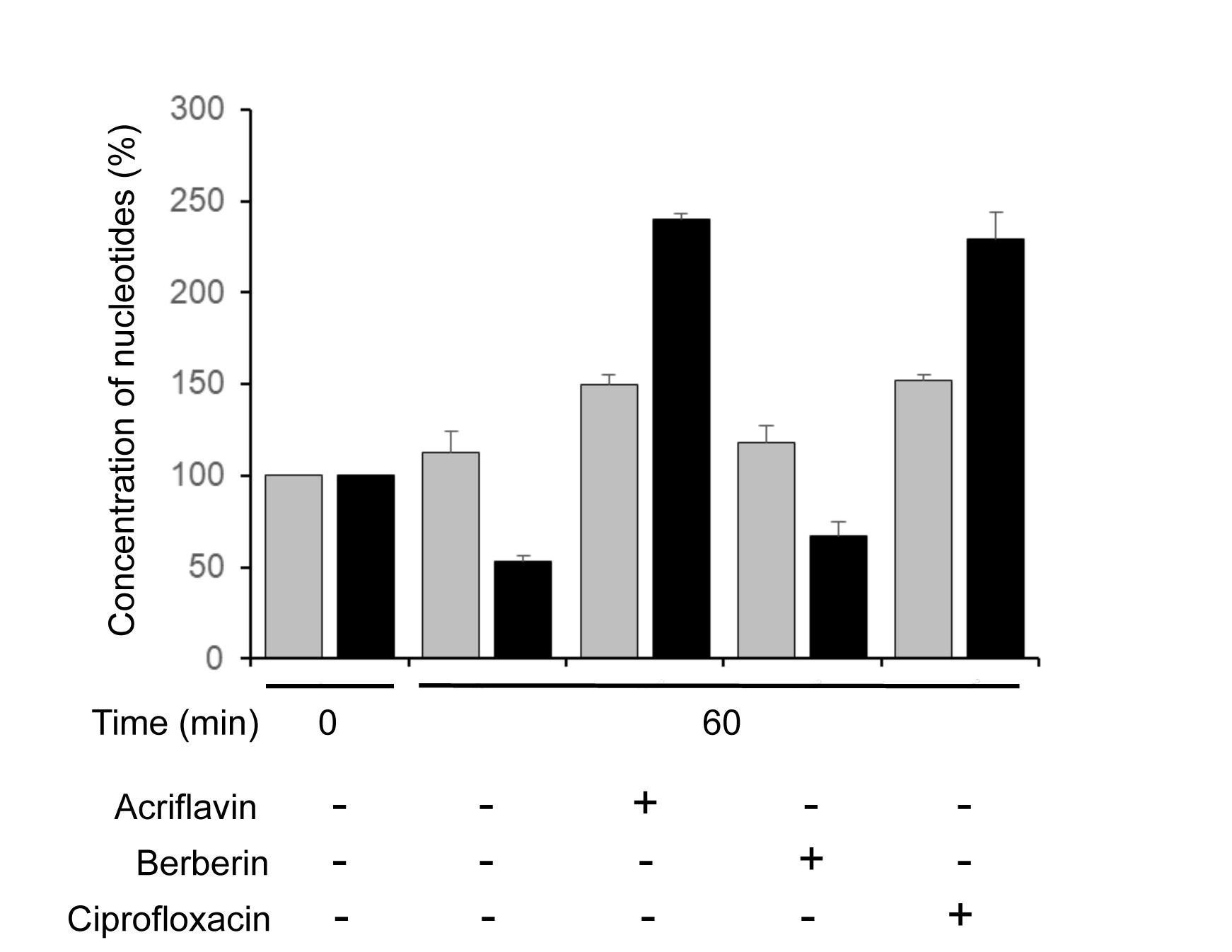


**Fig. S11.** **Intracellular concentrations of ATP and GTP following drug treatments.** *S. pneumonia* were grown in TH medium at 37 °C and when the absorbance at 600 nm reached 0.25, they were exposed to acriflavin (8 µg/mL), berberin (32 µg/mL) or ciprofloxacin (4 µg/mL) for 60 min. The cultures were rapidly filtered and frozen in liquid nitrogen before nucleotides extraction. Concentrations of ATP (grey bars) and GTP (black bars) were determined by mass spectrometry and were expressed in % of the non-treated control (T0). Error bars represent the standard error of the mean for four separate experiments.


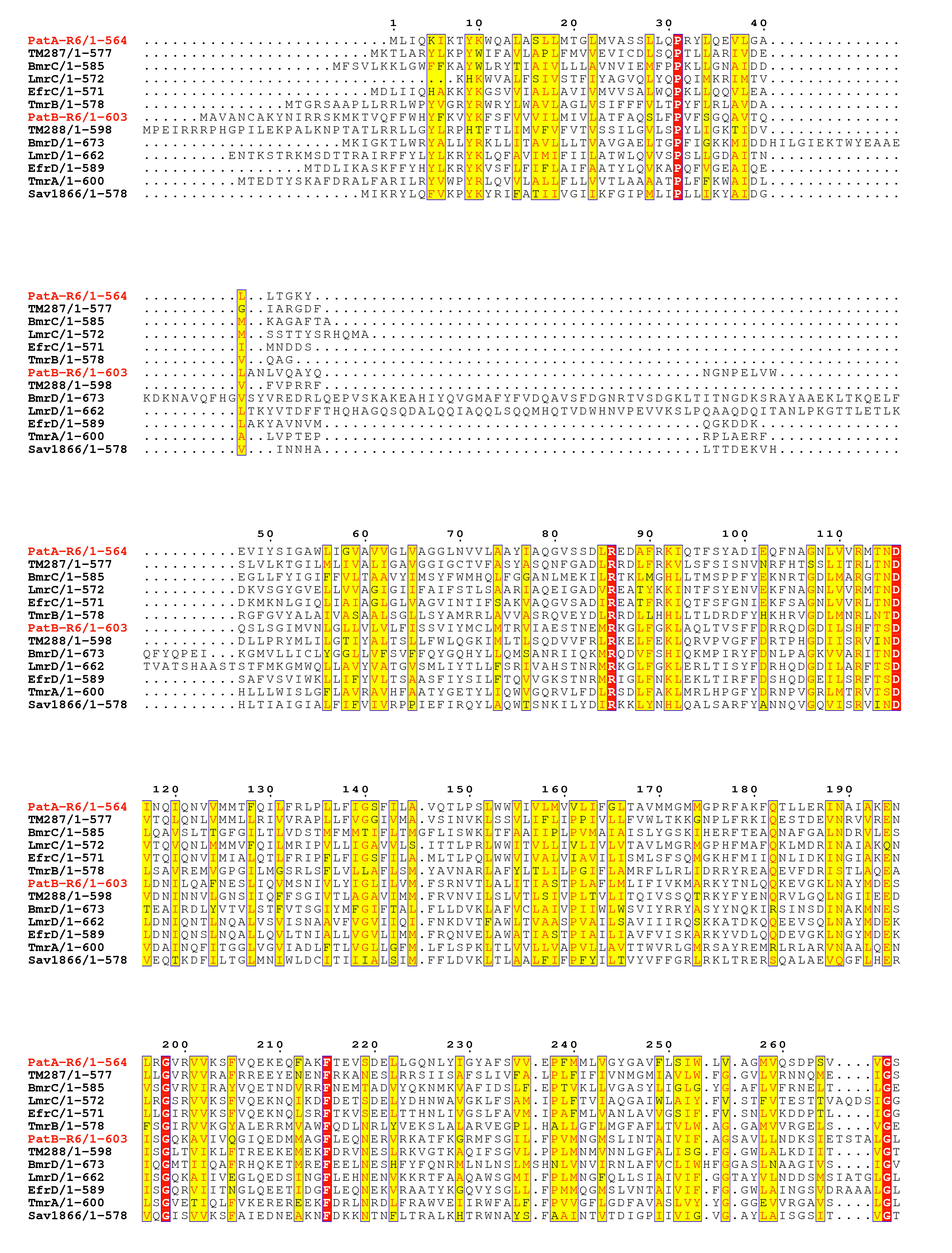


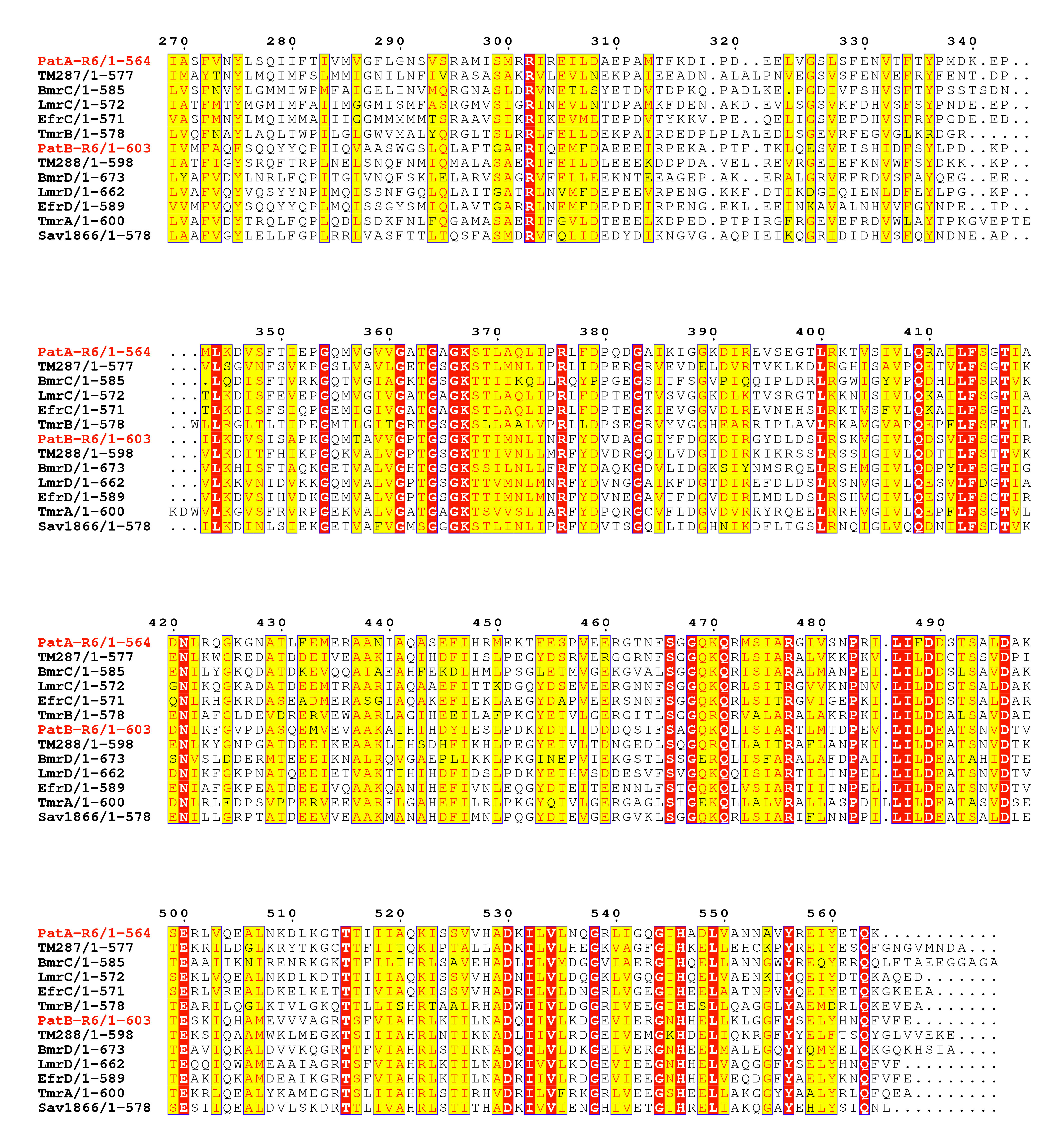


**Fig. S12. Sequence alignments of PatA/PatB with selected drug transporters from the ABC family.** The selected transporters include the heterodimeric TM287/TM288[^5^](#_ENREF_5), BmrC/BmrD[^6^](#_ENREF_6), LmrC/LmrD[^7^](#_ENREF_7), EfrC/EfrD[^8^](#_ENREF_8), TmrA/TmrB[^9^](#_ENREF_9), and the homodimeric Sav1866[^10^](#_ENREF_10). Alignments were performed using Jalview 2.10.1[^1^](#_ENREF_1) and the MUSCLE[^2^](#_ENREF_2) algorithm. The results are displayed with ESPript 3.0[^11^](#_ENREF_11). The sequences containing a degenerate nucleotide-binding domain are shown on the top of the alignment, while the sequences containing a consensual nucleotide-binding domain are shown on the bottom.

**REFERENCES**

1 Waterhouse, A. M., Procter, J. B., Martin, D. M., Clamp, M. & Barton, G. J. Jalview Version 2--a multiple sequence alignment editor and analysis workbench. *Bioinformatics* **25**, 1189-1191, doi:10.1093/bioinformatics/btp033 (2009).

2 Edgar, R. C. MUSCLE: multiple sequence alignment with high accuracy and high throughput. *Nucleic acids research* **32**, 1792-1797, doi:10.1093/nar/gkh340 (2004).

3 Epand, R. F., Savage, P. B. & Epand, R. M. Bacterial lipid composition and the antimicrobial efficacy of cationic steroid compounds (Ceragenins). *Biochim Biophys Acta* **1768**, 2500-2509, doi:10.1016/j.bbamem.2007.05.023 (2007).

4 Morein, S., Andersson, A., Rilfors, L. & Lindblom, G. Wild-type Escherichia coli cells regulate the membrane lipid composition in a "window" between gel and non-lamellar structures. *J Biol Chem* **271**, 6801-6809 (1996).

5 Hohl, M., Briand, C., Grutter, M. G. & Seeger, M. A. Crystal structure of a heterodimeric ABC transporter in its inward-facing conformation. *Nat Struct Mol Biol* **19**, 395-402, doi:10.1038/nsmb.2267 (2012).

6 Torres, C., Galian, C., Freiberg, C., Fantino, J. R. & Jault, J. M. The YheI/YheH heterodimer from Bacillus subtilis is a multidrug ABC transporter. *Biochim Biophys Acta* **1788**, 615-622 (2009).

7 Lubelski, J. *et al.* LmrCD is a major multidrug resistance transporter in Lactococcus lactis. *Molecular microbiology* **61**, 771-781, doi:10.1111/j.1365-2958.2006.05267.x (2006).

8 Hurlimann, L. M. *et al.* The Heterodimeric ABC Transporter EfrCD Mediates Multidrug Efflux in Enterococcus faecalis. *Antimicrobial agents and chemotherapy* **60**, 5400-5411, doi:10.1128/AAC.00661-16 (2016).

9 Noll, A. *et al.* Crystal structure and mechanistic basis of a functional homolog of the antigen transporter TAP. *Proceedings of the National Academy of Sciences of the United States of America* **114**, E438-E447, doi:10.1073/pnas.1620009114 (2017).

10 Dawson, R. J. & Locher, K. P. Structure of a bacterial multidrug ABC transporter. *Nature* **443**, 180-185, doi:10.1038/nature05155 (2006).

11 Robert, X. & Gouet, P. Deciphering key features in protein structures with the new ENDscript server. *Nucleic acids research* **42**, W320-324, doi:10.1093/nar/gku316 (2014).
